# Alterations of blood monocyte subset distribution and surface phenotype are linked to infection severity in COVID‐19 inpatients

**DOI:** 10.1002/eji.202149680

**Published:** 2022-05-06

**Authors:** David Haschka, Verena Petzer, Francesco Robert Burkert, Gernot Fritsche, Sophie Wildner, Rosa Bellmann‐Weiler, Piotr Tymoszuk, Guenter Weiss

**Affiliations:** ^1^ Department of Internal Medicine II Medical University of Innsbruck Innsbruck Austria; ^2^ Department of Internal Medicine V Medical University of Innsbruck Innsbruck Austria; ^3^ Data Analytics As a Service Tirol Innsbruck Austria

**Keywords:** COVID‐19, immunophenotyping, monocyte subsets, neutrophils, SARS‐CoV‐2

## Abstract

Severe coronavirus disease 19 (COVID‐19) manifests with systemic immediate proinflammatory innate immune activation and altered iron turnover. Iron homeostasis, differentiation, and function of myeloid leukocytes are interconnected. Therefore, we characterized the cellularity, surface marker expression, and iron transporter phenotype of neutrophils and monocyte subsets in COVID‐19 patients within 72 h from hospital admission, and analyzed how these parameters relate to infection severity. Between March and November 2020, blood leukocyte samples from hospitalized COVID‐19 patients (n = 48) and healthy individuals (n = 7) were analyzed by flow cytometry enabling comparative analysis of 40 features. Inflammation‐driven neutrophil expansion, depletion of CD16^+^ nonclassical monocytes, and changes in surface expression of neutrophil and monocyte CD64 and CD86 were associated with COVID‐19 severity. By unsupervised self‐organizing map clustering, four patterns of innate myeloid response were identified and linked to varying levels of systemic inflammation, altered cellular iron trafficking and the severity of disease. These alterations of the myeloid leukocyte compartment during acute COVID‐19 may be hallmarks of inefficient viral control and immune hyperactivation and may help at risk prediction and treatment optimization.

## Graphical abstract

Immunophenotyping of blood myeloid leukocytes in COVID‐19 inpatients and healthy control reveals four patterns of innate response involving nonclassical monocytes, CD86, CD163, CD40 and ferroportin (FPN1) surface expression in monocytes and neutrophil levels. The response patterns are linked to disease severity, systemic inflammation, and iron turnover.

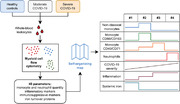

## Introduction

Since late 2019, the SARS‐CoV‐2 pandemic has resulted in a global health crisis. As of March 2022, more than 472 million infections and over 6.1 million COVID‐19 deaths were documented [1]. COVID‐19 is characterized by a heterogeneous clinical manifestation and divergent courses of the disease [2]. This ranges from asymptomatic infection, to ambulatory disease with wide‐ranged flu‐like complaints, up to severe respiratory or multiorgan failure, requiring hospitalization and treatment at intensive care unit [2, 3, 4]. Especially in the latter cases, an excessive overstimulation of the immune system, likewise driven by an impaired immune control of viral replication leads to a “cytokine release syndrome” with subsequent organ damage [5, 6]. Specifically, low levels of CD4^+^ and CD8^+^ cells, impaired antiviral type I IFN response and polymorphisms in the genes involved in antigen presentation and immune cell communication were associated with the risk of severe COVID‐19 [7, 8, 9, 10]. Furthermore, aberrant monocyte and neutrophil accumulation along with systemic inflammation and altered systemic iron homeostasis have been linked to the disease severity and a poor prognosis [11, 12, 13, 14, 15, 16, 17, 18, 19, 20]. This pertains to the key role of myeloid leukocytes, that is, macrophages, monocytes, and neutrophils in the SARS‐CoV‐2 pathogen control and orchestration of the inflammatory response [13, 16, 17, 21–24]. In addition, monocytes or macrophages play an important role in the orchestration of iron homeostasis, and inflammatory processes result in macrophage or monocyte iron retention, whereas iron exerts distinct effects on macrophage polarization and immune effector functions [25, 26, 27].

Human blood monocytes consist of three populations: the major subset of CD14^+/high^ CD16^−/low^ classical cells, and substantially less abundant CD14^+/high^ CD16^+/high^ intermediate, and CD14^−/low^ CD16^+/high^ nonclassical monocytes [28]. These subsets display differences in the pattern to activate stimuli and immune effector functions such as phagocytosis, antigen presentation, and cytokine production [28]. Alterations in the monocyte subset distribution and surface molecule repertoire were observed in multiple inflammatory conditions, including COVID‐19, and were proposed to correlate with the disease course and prognosis [11, 15–18, 21–23, 28–33].

From the clinical and immunological point of view, there is an ongoing search for markers characterizing the clinical course of COVID‐19 as well as the protective and pathological immune responses to the pathogen. Herein, we investigated the surface molecule repertoire of myeloid innate immune cells from hospitalized COVID‐19 patients by flow cytometry and linked the surface phenotype to the disease severity and patterns of systemic inflammation and iron turnover by self‐organizing map (SOM) clustering [34, 35].

## Results

### Study cohort

**Figure 1:**
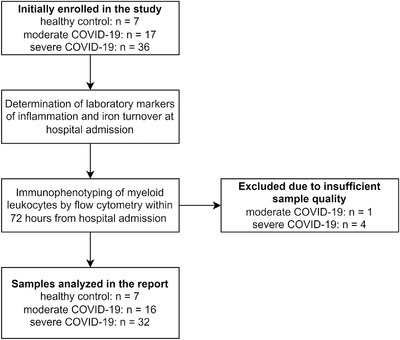
Analysis inclusion scheme. Numbers of individuals initially enrolled in the study, excluded from the analysis, and included in the final analysis cohort are indicated.

The study was conducted between March and November 2020 and encompassed healthy controls (n = 7) and hospitalized COVID‐19 patients (n = 53 enrolled) whose blood myeloid compartment was analyzed by flow cytometry within 72 h from hospital admission (Supporting information Figures S1 and S2). Due to insufficient flow cytometry sample quality, five COVID‐19 patients were excluded from further analysis. Finally, samples from seven healthy controls, 16 moderate COVID‐19 patients not requiring supplemental oxygen therapy during hospitalization (WHO score 3), and 32 severe COVID‐19 individuals requiring low/high flow oxygen treatment or mechanical ventilation (WHO score 4–6) were available for the analysis (Figure 1). Among severe COVID‐19 cases, 22% were treated at an intensive care unit and 6.2% deceased during hospitalization. Males constituted 56% of the moderate and 72% of the severe COVID‐19 group, but the sex distribution difference was not significant. The median age was significantly different between the healthy controls (36 [interquartile range [IQR]: 32–42] years), moderate (46 [IQR: 38–60] years), and severe COVID‐19 patients (71 [IQR: 64–79], years) and peaked in the severe COVID‐19 group (Supporting information Table S1).

### Sustained systemic and cellular inflammatory response and iron restriction in COVID‐19

Systemic inflammation, as evidenced by elevated levels of IL6, C‐reactive protein (CRP), and neopterin, was present in hospitalized COVID‐19 individuals and was linked to poor outcomes [12, 14–16]. In our study cohort, plasma IL6, CRP, and neopterin concentrations at admission were significantly higher in patients developing severe COVID‐19 than in subjects with moderate disease, as described previously [12] (Supporting information Figure S3A). These differences in inflammatory markers were paralleled by significantly reduced circulating iron concentrations, transferrin saturation (TF‐Sat), and elevated circulating ferritin levels in severe COVID‐19 as compared with the moderate group (Supporting information Figure S3B). Such a phenomenon reflects iron restriction in response to a severe infection, as reported previously for COVID‐19 [16, 20, 36–39].

### Alterations of the myeloid compartment composition and surface phenotype in healthy volunteers and moderate and severe COVID‐19 patients

Next, we investigated how this systemic inflammatory and iron‐restricted milieu shapes the composition, inflammatory, and iron transporter protein repertoire of myeloid leukocytes [26, 40]. To this end, we analyzed the whole blood monocyte and neutrophil compartments of healthy controls and COVID‐19 patients within 72 h of hospital admission with multicolor flow cytometry (Supporting information Table S2). Neutrophils were defined within the lymphocyte lineage‐negative leukocyte population (Lin^−^: CD3^−^ CD19^−^ CD56^−^) by logical gating (AND) of the CD16^+^, CD11b^+^, CD62L^+^, and SSC^hi^ cells. Monocytes were identified within the non‐neutrophil gate by UMAP clustering (uniform manifold approximation and projection) [41] in respect to CD11b, CD14, CD15, CD16, CD62L, CCR2, CX3CR1, and HLA‐DR expression levels (Supporting information Figure S1). Classical, intermediate, and nonclassical monocyte subsets were defined by differences in CD14, CD16, HLA‐DR, CCR2, and CX3CR1 surface levels [28] within the monocyte cluster cells (Supporting information Figure S2). Of note, the additional markers used to define monocyte populations (HLA‐DR, CCR2, and CX3CR1) displayed similar expression differences between classical, intermediate, and nonclassical cells from healthy donors and COVID‐19 patients. No downregulation of HLA‐DR levels in classical monocytes, described previously [11, 16, 17], could be observed in COVID‐19 patients as compared with healthy controls (Supporting information Figure S4). In turn, neutrophil CD14 expression was found significantly different between the study groups and was the highest in moderate COVID‐19 individuals (Supporting information Figure S5).

**Figure 2:**
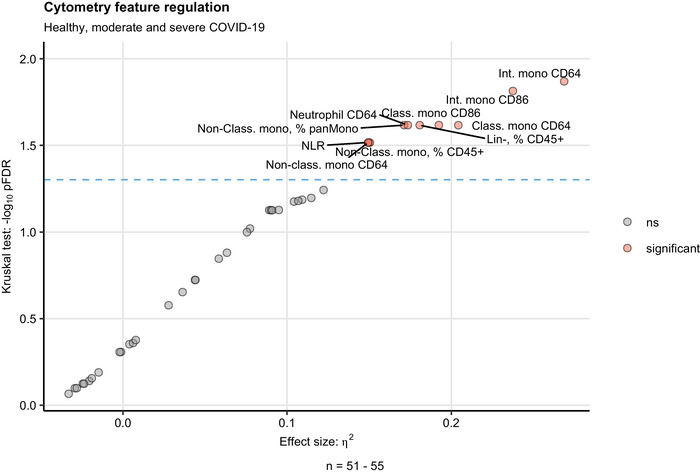
Screening of the flow cytometry features for the differences between healthy controls, moderate and severe COVID‐19. Levels of 40 parameters of monocytes and neutrophils were measured by flow cytometry in healthy controls, moderate and severe COVID‐19 patients within 72 h posthospital admission (Supporting information Table S3). The parameter levels were compared between healthy controls, moderate and severe COVID by Kruskal–Wallis test corrected for multiple testing with Benjamini–Hochberg method (false discovery Rate, FDR). Significance and $\eta^{2}$ effect size statistic is presented in the plot. Each point represents a single flow cytometry feature. Significant features are highlighted in read and labeled with their names. The horizontal dashed line represented the significance cutoff (*p* = 0.05). N = 51–55 biological replicates (blood cell donors) per cytometry parameter.

A total of 40 features could be measured with our flow cytometry pipeline including the percentages of neutrophils, classical, intermediate, and nonclassical monocytes as well as isotype staining‐controlled surface levels of pro‐ and anti‐inflammatory markers, iron‐import protein CD71 (transferrin receptor 1), and iron exporter FPN1 (ferroportin) (Supporting information Table S3). Among them, 10 variables displayed significant differences between healthy controls and moderate and severe COVID‐19 cell donors as determined by Kruskal–Wallis test: (1) percentage of lineage‐negative cells, (2) neutrophil:lymphocyte ratio (NLR), (3) percentages of nonclassical monocytes within the CD45^+^ compartment and (4) pan‐monocytes, expression levels of CD64 in (5) neutrophils, (6) classical, (7) intermediate and (8) nonclassical monocytes as well as surface CD86 levels in (9) classical and (10) intermediate monocytes (Figure 2).

**Figure 3:**
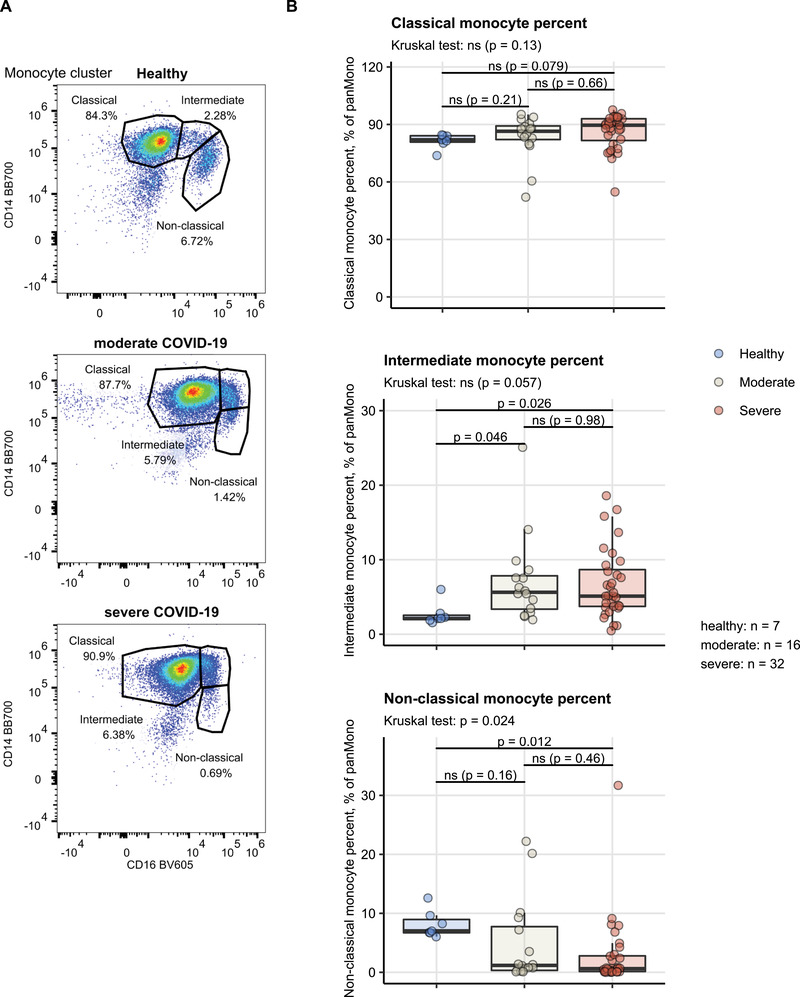
Monocyte subset distribution in healthy controls, moderate and severe COVID‐19. Percentages of classical, intermediate, and nonclassical monocytes within the monocyte cluster cells (for gating strategy see Supporting information Figures S1 and S2) were measured in healthy controls, moderate and severe COVID‐19 patients. Statistical significance was determined by Kruskal–Wallis test with Mann–Whitney post‐hoc test. Testing results were adjusted for multiple comparisons with Benjamini–Hochberg method. (A) Representative cytometry result of one healthy, one moderate COVID‐19, and one severe COVID blood cell donor. Monocyte cluster cells are presented. (B) Summary plots. Kruskal–Wallis *p* values are indicated in the plot subheading, post‐hoc test results are shown in the plot, numbers for complete observations are presented next to the plot. Each point represents a single observation, boxes represent medians with interquartile range (IQR), whiskers span over the 150% IQR range. N = 55 biological replicates (blood cell donors, healthy: n = 7, moderate COVID‐19: n = 16, severe COVID‐19: n = 32).

More specifically, an expansion of neutrophils at the cost of Lineage‐positive lymphocytes could be observed in severe COVID‐19, resulting in substantially higher NLR values (median: 2.8 [IQR: 2–5.3]) as compared with moderate COVID‐19 (1.9 [IQR: 0.82–2.6]) or healthy controls (1.6 [IQR: 1.3–2.3]) (Supporting information Figure S6). This phenomenon was accompanied by a depletion of the nonclassical monocyte subset, which was evident in moderate COVID (median % of pan‐monocytes: 1.2 [IQR: 0.33– 7.7]) and significant in the severe disease (1.2 [IQR: 0.33–7.7]) as compared with healthy cell donors (7 [IQR: 6.7–9]). In addition, the intermediate monocyte compartment was substantially, yet not significantly, larger in COVID‐19 patients (median, % of CD45^+^ cells, moderate: 5.6 [IQR: 3.4–7.8], severe: 5.1 [IQR: 3.7–8.7]) than in control individuals (2.1 [IQR: 2–2.6]) (Figure 3). Of note, neutrophil expansion [14, 16–19] along with a profound reduction of nonclassical monocyte levels [11, 15–18, 21–23, 31–33] has been described as a characteristic of acute COVID‐19.

The neutrophil CD64 was virtually absent in healthy controls (median ΔMFI: 180 [IQR: 66–700]) but highly expressed in moderate (1000 [IQR: 730–2000]) and severe COVID‐19 (2600 [IQR: 980–4300]). A similar pattern of CD64 regulation was discerned for classical (median ΔMFI, healthy: 42 000 [IQR: 35 000–45 000], moderate: 81 000 [IQR: 45 000–150 000], severe: 110 000 [IQR: 68 000–120 000]), and intermediate monocytes (median ΔMFI, healthy: 23 000 [IQR: 19 000–25 000], moderate: 61 000 [IQR: 33 000–86 000], severe: 85 000 [IQR: 65 000–110 000]), which, however, expressed this FCγ receptor at high levels also in noninfected controls (Supporting information Figure S7). Surface expression of CD86 in classical (median ΔMFI, healthy: 6100 [IQR: 4500–6700], moderate: 10 000 [IQR: 9300–17 000], severe: 8400 [IQR: 5700–11 000]), and intermediate monocytes (healthy: 9500 [IQR: 8200–12 000], moderate: 19 000 [IQR: 15 000–25 000], severe: 16 000 [IQR: 11 000–19 000]) reached the maximum in moderate COVID‐19 patients as compared with healthy controls and severe COVID‐19 patients (Supporting information Figure S8).

### Patterns of innate myloid leukocyte response in acute COVID‐19

To elucidate possible patterns of coregulation of myeloid leukocyte quantity and surface phenotype, we subjected the set of 40 flow cytometry parameters measured in our study (Supporting information Table S3) and the healthy and COVID‐19 study participants to SOM clustering [34, 35].

**Figure 4:**
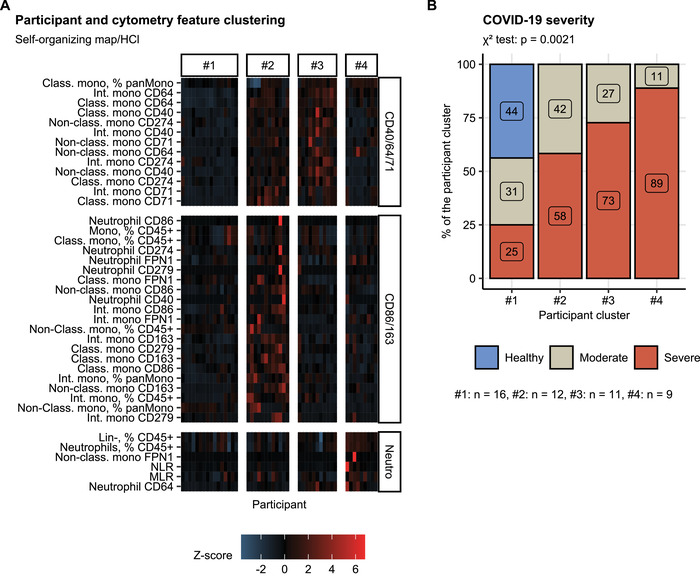
Unsupervised clustering of healthy controls and COVID‐19 individuals by myeloid leukocyte cytometry features. Flow cytometry features (n = 40, Supporting information Table S3) and study participants (n numbers presented in B) were subjected to clustering analysis with a combined self‐organizing map (SOM)–hierarchical clustering (HCl) algorithm (Supporting information Figure S9). Three clusters of cytometry features termed after the most characteristic features (neutrophil, CD40, CD64, CD71, CD86, and CD163) and four participant clusters were identified. (A) Normalized levels of flow cytometry parameters (Z‐score) in the participant (horizontal) and cytometry feature clusters (vertical plot facets) presented as a heat map. N = 48 biological replicates (blood cell donors, cluster #1: n = 16, #2: n = 12, #3: n = 11, #4: n = 9). (B) Percentages of healthy controls, moderate and severe COVID‐19 participants in the participant clusters. Statistical significance was determined by $\chi^{2}$ test. N = 48 biological replicates (blood cell donors, cluster #1: n = 16, #2: n = 12, #3: n = 11, #4: n = 9).

By this means, we could identify three clusters of the cytometry features and four clusters of the study individuals (Supporting information Figure S9). The cytometry feature clusters included (1) a subset consisting primarily of markers of neutrophil expansion (termed “Neutro”: Lin^−^ and neutrophil levels, neutrophil CD64, NLR, and monocyte:lymphocyte ratio(MLR)), (2) a cluster dominated by CD86, CD163, and FPN1 expression in neutrophils and monocyte subpopulations and monocyte subset quantity variables (termed: “CD86/CD163”), and (3) a cluster encompassing mainly D40, CD71, and CD64 levels in myeloid populations (termed “CD40/CD64/CD71”) (Figure 4A).

Healthy controls and moderate COVID‐19 cases comprised 75% of the largest participant cluster #1 which was characterized by the lowest levels of CD40/CD64/CD71 parameters as well as healthy‐like monocyte subset distribution, neutrophil levels, and neutrophil CD64 expression (Figure 4A,B). Interestingly, none of the severe COVID‐19 cases assigned to the cluster #1 required ICU treatment (Supporting information Table S4). The remaining clusters consisted predominantly of severe COVID‐19 patients with increasing frequency. The hallmark of the cluster #2 was an increased expression of CD86, C163, the immunosuppressive PD‐L1 molecule (CD279), and the iron exporter FPN1 in classical and intermediate monocytes as well as an expansion of the intermediate subset. The participant cluster #3 in turn was primarily characterized by an elevated neutrophil and monocyte CD64 as well as a depletion of the nonclassical monocytes. Finally, leukocyte samples assigned to the cluster #4 displayed a consistent expansion of the neutrophil compartment, elevated CD64 in neutrophils, and a depletion nonclassical monocytes (Figure 4A).

In comparative analysis of COVID‐19 individuals, the participant clusters demonstrated similar age and body mass index. In turn, the clusters #1 and #4 COVID‐19 individuals were predominantly males (78%) as compared with the remaining subjects. Concerning the disease course, the fraction of participants requiring respiratory support was the lowest in cluster #1 and peaked in the clusters #3 and #4. However, none of these clinical features was significantly different in the all‐group and the cluster #1 versus rest comparisons (Supporting information Table S4).

**Figure 5:**
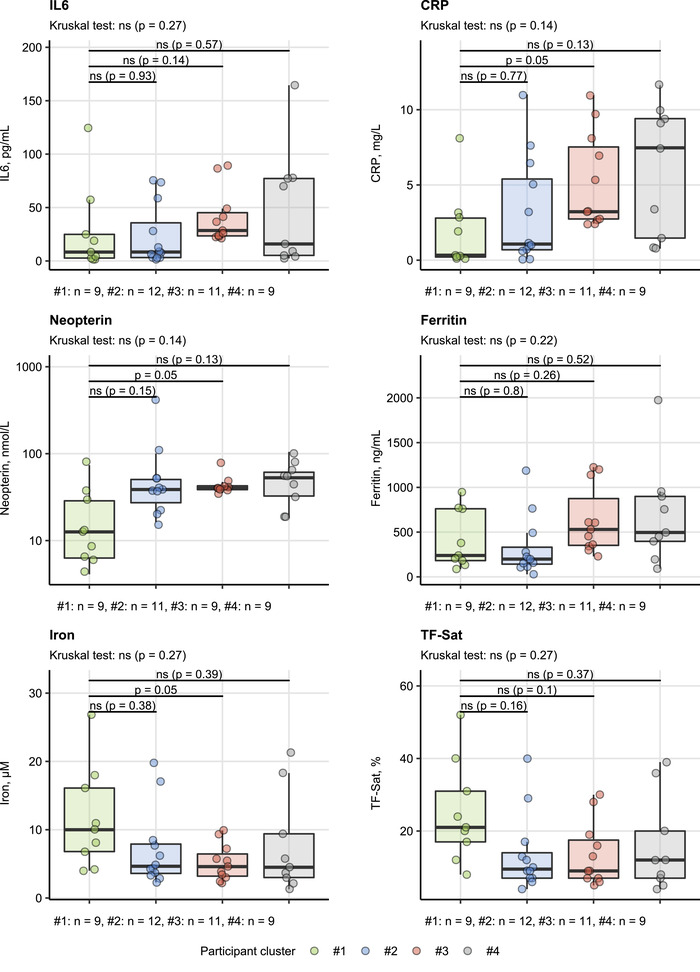
Differences in systemic inflammation status between the clusters of study participants. Blood levels of markers of inflammation (IL6: interleukin 6, C‐reactive protein: CRP, neopterin) and iron turnover (ferritin, plasma iron, TF‐Sat: transferrin saturation) were compared in COVID‐19 patients assigned to the participant clusters (Figure 4A, Supporting information Figure S9). Statistical significance was determined by Kruskal–Wallis test with Mann–Whitney post‐hoc test. Testing results were adjusted for multiple comparisons with Benjamini–Hochberg method. N = 38–41 biological replicates (blood cell donors) per inflammatory parameter. Numbers of biological replicates per cluster are presented under each plot.

Finally, we investigated the inflammatory status and iron turnover parameters in COVID‐19 patients assigned to the participant clusters. Although the differences between the clusters did not reach statistical significance following multiple testing correction, a clear tendency toward increased blood levels of IL6, CRP, and neopterin was observed in the clusters #2, #3, and #4 as compared with the healthy‐like cluster #1 (Figure 5, Supporting information Table S4). Accordingly, the clusters #2, #3, and #4 had the lowest levels of circulating iron and TF‐Sat values suggestive of inflammatory iron restriction [36]. Of interest, despite the elevated inflammatory marker levels, blood ferritin concentration in the cluster #2 tended to be lower than in the similarly inflammatory clusters #3 and #4 (Figure 5, Supporting information Table S4).

Collectively, the clustering analysis results suggest the presence of qualitatively and quantitatively distinct phenotypes of innate myeloid cell response to SARS‐CoV‐2 infection. Such phenotypic diversity is likely associated with COVID‐19 severity and partially independent of the demographic patient background.

## Discussion

Exaggerated cytokine and innate cell response is hallmark of severe COVID‐19, requiring hospitalization, oxygen, or ICU therapy [11, 12, 14–19]. In agreement with previous reports, we observed elevated circulating IL6, CRP, and neopterin levels and an expansion of neutrophils with a concomitant reduction in lymphocytes in COVID‐19 patients requiring respiratory support. Similar to other infections [36, 38], this resulted in reduced levels of circulating iron and elevated ferritin, in line with few published reports on COVID‐19 [16, 20, 27, 37, 37, 39]. The sole cellular iron exporter, FPN1, in monocytes and macrophages, is downregulated by the hormone hepcidin and diverse inflammatory cytokines and upregulated by cellular iron [25, 36, 42, 43]. Interestingly, hypoferremia in acute COVID‐19 was accompanied by only mildly elevated blood hepcidin levels [37, 39], which may explain the consistently elevated FPN1 levels in the subsets of inflammatory COVID‐19 patients assigned to the participant cluster #2. In general, the changes in the cellular iron trafficking and systemic turnover in COVID‐19 may bear relevance for the effective SARS‐CoV‐2 control because iron levels significantly impact on the differentiation of T cells and monocytes and on macrophage polarization and also affect antimicrobial immune effector mechanisms [25, 36]. Furthermore, intracellular iron availability may promote viral replication [44]. As such, the interplay of these processes requires further mechanistic and clinical investigations.

By applying flow cytometry, we were able to characterize a palette of 40 variables associated with the quantity, pro‐, anti‐inflammatory, and iron transport phenotype of neutrophils, classical, intermediate, and nonclassical monocytes. Among them, a consistent depletion of nonclassical monocytes, in part paralleled by an expansion of the intermediate subset became evident in COVID‐19 patients. This phenomenon was also described by others and suggested to aggravate with infection severity[11, 15, 16, 17, 18, 21, 22, 23, 31, 32, 33]. It was also proposed to predict the risk of critical disease and respiratory failure [17, 21, 23]. The CD16^+^ nonclassical subset contraction was suggested to be specific for COVID‐19 but not for bacterial sepsis [16]. However, results of an experimental endotoxin challenge in humans demonstrated a general depletion of blood monocytes and a step‐wise reconstitution involving immediately the classical population, followed by the remaining subsets [30]. Hence, the nonclassical monocyte contraction in COVID‐19 may reflect an early step in the nonspecific inflammatory response to SARS‐CoV‐2. A subsequent repopulation of the nonclassical compartment by classical monocyte‐derived precursors during convalescence can be inferred from longitudinal COVID‐19 immunophenotyping studies [16, 17, 18].

Our immune‐phenotyping results revealed an upregulation of the FC‐γ receptor CD64 in neutrophils, classical and intermediate monocytes in COVID‐19 patients as described recently [11, 17, 19, 32, 45]. For neutrophils, the elevated CD64 expression together with lowered neutrophil CD14 levels observed in the study group of severe COVID‐19 patients may indicate the presence of less mature proneutrophil‐like CD64^+^ CD14^low^ cells expanding in early COVID‐19 [11] and sepsis [46, 47], and hence, represent a general reaction to a strong inflammatory stimuli triggering emergency myelopoiesis [11, 17]. This is further supported by our clustering data, where the high neutrophil CD64 levels co‐occurred with the neutrophil expansion in the highly inflammatory, severe COVID‐19‐dominated participant cluster #4. Since the CD64 expression in neutrophils and monocytes was the highest in the clusters #3 and #4 comprising of predominantly ventilated severe COVID‐19 patients, secondary bacterial airway coinfections may also contribute to the upregulation of this marker. The expression of CD86 in the intermediate and classical monocyte subsets followed a distinct regulatory pattern with a peak in moderate COVID‐19. which is in line with other publications [14, 22]. In the clustering analysis of our cytometry data, monocyte CD86 expression was found to be coregulated with the immunosuppressive ligand CD279 (PD‐L1), the alternative macrophage activation marker CD163 and the iron exporter FPN1, the latter being highest in the participant cluster #2. Collectively, this suggests that in a fraction of COVID‐19 patients, subsumed under the participant cluster #2 in the study cohort, the blood monocytes may acquire an immunoregulatory, T‐cell suppressive, iron export CD86^high^ CD279^high^ FPN1^high^ phenotype. Notably, expansion of a similar HLA‐DR^low^, CD86^hi^, CD163^hi^, and T cell‐suppressive classical monocyte populations was described for moderate and severe COVID‐19 [11, 16–18, 24]. In our cohort, however, no significant differences in monocyte HLA‐DR expression between COVID‐19 patients and healthy controls could be observed, which may be attributed to the differences in the detection methodology (multicolor flow cytometry versus CyTOF or spectral flow cytometry).

To get a thorough overview of myeloid leukocyte response in acute COVID‐19, we performed a clustering analysis with the SOM technique [34, 35], by which we could identify four distinct clusters among the study participants. The largest, healthy‐like cluster #1 was associated with near‐normal composition and surface phenotype of neutrophils and monocytes as well as the lowest levels of systemic inflammation, largely normal iron homeostasis and low risk of ICU admission. The other clusters were characterized by a clearly inflammatory and iron‐restrictive phenotype but differed in the myeloid leukocyte characteristic. In particular, the cluster #2 was hallmarked by the presence of CD86‐, CD163‐, CD279‐, and FPN1‐expressing monocytes suggestive of an immunoregulatory phenotype [11, 16–18, 24] as discussed above. The cluster #3 was characterized primarily by high levels of monocyte CD40 and CD64 which may reflect a proinflammatory priming of those cells, as observed in chronic inflammatory conditions [48, 49, 50]. Finally, the expansion of CD64‐expressing neutrophils in the participant cluster #4 may represent an inflammation‐triggered emergency myelopoiesis and occurrence of immature neutrophils in the circulation of severe COVID‐19 patients [11, 17, 46, 47]. The prognostic and therapeutic relevance of such individual heterogeneity of the innate response to the SARS‐CoV‐2 pathogen needs to be investigated in a larger study collective.

Our study bears limitations referring to a small number of participants which, together with the lack of verification cohort, precluded more systematic analyses adjusted for known risk factors of severe COVID‐19 such as age, sex, and comorbidity. The alterations in neutrophil and monocyte subset quantities and phenotype as well as the clinical utility of the predictive myeloid score needs to be verified in a larger follow‐up study, possibly including longitudinal determinations to capture dynamics of the myeloid leukocyte compartments [15–18, 22]. Additionally, differences in age structure and likely in the frequency of comorbidity between the healthy controls and COVID‐19 patients in the study cohort may have confounded the presented analysis results.

Our study corroborates the massive changes in the neutrophil and monocyte compartments in acute moderate and severe COVID‐19 including expansion of neutrophils, depletion of the nonclassical monocytes, as well as regulation of CD64 and CD86 expression and altered iron trafficking in myeloid leukocytes. Finally, we demonstrate the presence of four patterns of the innate myeloid response to SARS‐CoV‐2 with possible implications for diagnosis, risk assessment, and therapy of COVID‐19.

## Materials and methods

### Study population and procedures

The study was performed between March 21, 2020 and November 16, 2020 at the hospital of the University of Innsbruck, Tyrol, Austria. The inclusion criteria for COVID‐19 participants were age ≥18 years and hospital admission because of acute SARS‐CoV‐2 infection confirmed by nasal or oral swab PCR test. The exclusion criterion was a critical physical or mental state at admission, precluding giving informed study participation consent. Healthy controls were recruited among the institution's health care workers.

Measurement of the laboratory inflammation markers: CRP, IL6, neopterin, and iron status parameters: ferritin, iron concentration, and TF‐Sat, in the blood at admission was done as a routine determination at the hospital central laboratory. Whole venous EDTA blood (5–10 mL) for the flow cytometry measurement was withdrawn within 72 h from hospital admission.

### Measurement of laboratory inflammation and iron markers

CRP in plasma was determined by a particle‐enhanced immunological turbidity assay: human CRP agglutinated with latex particles coated with anti‐CRP mAb (mouse). Aggregates were determined turbidimetrically. IL‐6 was assessed in plasma by an electrochemiluminescence assay in the sandwich principle. Neopterin was analyzed by a competitive enzyme immunoassay for the direct quantitative determination of neopterin. Quantitative determination of ferritin in human plasma was performed using a latex particle‐enhanced immunological turbidity assay. The amount of iron in human plasma was measured photometrically by the FerroZine method without deproteinization. TF‐Sat was measured by an immunological turbidity test.

### Flow cytometry

The flow cytometry staining was done with the antibodies against the qualitative backbone markers of the lymphocyte, neutrophil, and monocyte lineages and the test antibodies for qualitative markers of pro‐ and anti‐inflammatory surface phenotype (CD40, CD64, CD80, CD86, CD163, CD274/PD‐L1, and CD279/PD‐1) and iron turnover proteins (CD71/transferrin receptor 1 and FPN1/ferroportin) [42, 51] listed in Supporting information Table S2. The antibodies were purchased from Beckton–Dickinson (Franklin Lakes, NJ), Thermo Fisher (Waltham, MA), or Biolegend (San Diego, CA).

Whole blood was subjected to RBC depletion with ammonium‐chloride‐potassium buffer, followed by extensive washing with FACS buffer (1% FCS, 0.5 mM EDTA in PBS) and staining with the backbone antibody mix (dilutions: 1:20–1:200 in BD Horizon Brilliant Stain Buffer, Beckton–Dickinson) for 10 min at 4°C. Next, the sample was washed, split, and stained with the separate isotype or test antibody mix (1:200 dilution in BD Horizon Brilliant Stain Buffer) for an additional 10 min at 4°C. The samples were measured with a Cytoflex S device (Beckman Coulter, Brea, CA) and analyzed with FlowJo 10.7 software (Beckton–Dickinson).

The cytometry data were analyzed by an experienced operator blinded to the study group assignment with a strategy depicted in Supporting information Figures S1 and S2 according to flow cytometry guidelines [52]. Neutrophils were identified within the lineage‐negative leukocytes (CD3^−^ CD19^−^ CD56^−^) by logical gating (AND) of CD11b^+^, CD16^+^, CD62L^+^, and SSC^lo^ events. Monocytes were defined within the non‐neutrophil gate (logical NOT gating) by UMAP [41] in respect to CD11b, CD14, C15, CD16, CD62L, CCR2, CX3CR1, and HLA‐DR expression. The monocyte cluster was identified by high CD14, CCR2, CX3CR1, and HLA‐DR levels. Classical, intermediate, and nonclassical monocytes were identified within the monocyte UMAP cluster by differences in CD14, CD16, CCR2, CX3CR1, CD62L, and HLA‐DR expression [28]. Surface expression was expressed as a difference in median signal intensity (ΔMFI) between the specific staining and the isotype. Neutrophil CD71 and CD163 as well as CD80 staining in neutrophils and monocytes were excluded from the downstream analysis due to low and virtually invariant expression levels. MLR was calculated as a ratio of the CD45^+^ percent of HLA‐DR^+^ pan‐monocytes to Lineage^+^ (CD3, CD19, CD56) cells. NLR was calculated as a ratio of the CD45^+^ percent of CD15^+^ SSC^hi^ neutrophils to Lineage^+^ cells.

### Statistical analysis

Statistical analysis was done with R version 4.0.5 and packages, *tidyverse*, *DescTools*, *rstatix* and *cowplot* packages [53, 54], the in‐house‐developed packages *ExDA* (explorative data analysis and hypothesis testing, https://github.com/PiotrTymoszuk/ExDA) and *clustTools* (wrappers for clustering and SOMs, https://github.com/PiotrTymoszuk/clustTools).

Since most of the analyzed variables demonstrated non‐normal distribution (Shapiro–Wilk test), nonparametric hypothesis tests were utilized in the downstream analyses. To search for cytometry features (Supporting informationn Table S3) significantly differing between healthy controls, moderate and severe COVID‐19 patients, Kruskal–Wallis test with with Benjamini–Hochberg (FDR: False Discovery Rate) correction for multiple comparisons was applied [55]. The significant variables were defined by the pFDR < 0.05 cutoff. Post‐hoc testing for pairwise differences between the groups was accomplished by Benjamini–Hochberg corrected Mann–Whitney U test. Clustering of the flow cytometry features (Supporting information Table S3) and of the healthy and COVID‐19 study participants in respect to the flow cytometry features was done with a combined SOM and hierarchical clustering (HCl) procedure [34, 35]. The data were preprocessed by *Z*‐score normalization and the participants with missing values eliminated. The cytometry features or participants were subjected to dimensionality reduction by SOM (5 × 5 hexagonal grid, cosine distance between the features/participants) and, subsequently, the SOM nodes were clustered with the HCl (cosine distance between the nodes, Ward D2 algorithm). The choice of the SOM node cluster number was motivated by the bend of the within‐cluster sum‐of‐square curve and a visual analysis of the dendrograms (Supporting information Figure S9). The fraction of clustering variance (ratio of the total within‐cluster to total sum of squares) was 0.38 and 0.58 for the feature and participant clustering, respectively.

## Ethics approval

The study was performed in accordance with the Declaration of Helsinki and the European Data Policy and approved by the institutional review board of the University of Innsbruck (approval number: 1091/2020).

## Patient consent

All study participants were conscious and gave a written informed consent prior to enrollment. The participants’ data were anonymized after recording hence making identification of the particular subjects impossible.

## Author's contribution

Conceptualization: DH, VP, DW, PT, GW; data curation: DH, VP; formal analysis: DH, PT; investigation: DH, VP, FB, GF, SW, RB, PT; methodology: DH, VP, PT; resources: DW, GW; visualization: DH, VP, PT; original draft preparation: DH, PT; review and editing: DH, VP, FB, GF, SW, RB, DW, PT, GW.

## Conflict of interest

PT now owns his data science enterprise, Data Analytics as a Service Tirol. The author declares no other conflict of interest. Other authors declare that non conflict of interest exists.

### Peer review

The peer review history for this article is available at https://publons.com/publon/10.1002/eji.202149680.

**Abbreviations**
CD71: transferrin receptor 1
CRP: C‐reactive protein
FDR: False Discovery Rate
FPN1: ferroportin
HCl: hierarchical clustering
IQR: interquartile range
MLR: monocyte:lymphocyte ratio
NLR: neutrophil:lymphocyte ratio
SOM: self‐organizing map
TF‐Sat: transferrin saturation
UMAP: uniform manifold approximation and projection

## Supporting information



Supporting Information

Supporting Information

## Data Availability

The data that supports the findings of this study are available in the supplementary material of this article. The R analysis pipeline is available at https://github.com/PiotrTymoszuk/covFacs. The data set with variables analyzed in the study is available as Supporting information Table S5.

## References

[1] WHO. Weekly operational update on COVID‐19. Emergency Situational Updates. 2021: 1–10. Available at: https://www.who.int/publications/m/item/weekly-operational-update-on-covid-19–22-february-2022.

[2] Burkert, F. R. , Lanser, L. , Bellmann‐Weiler, R. and Weiss, G. , Coronavirus disease 2019: clinics, treatment, and prevention. Frontiers in Microbiology. 2021. 12: 761887 34858373 10.3389/fmicb.2021.761887 PMC8631905

[3] Sahanic, S. , Tymoszuk, P. , Ausserhofer, D. , Rass, V. , Pizzini, A. , Nordmeyer, G. , Hüfner, K. et al., Phenotyping of acute and persistent COVID‐19 features in the outpatient setting: exploratory analysis of an international cross‐sectional online survey. Clinical Infectious Diseases. 2021. ciab978. 10.1093/CID/CIAB978 PMC8767855 34849652

[4] Oberfeld, B. , Achanta, A. , Carpenter, K. , Chen, P. , Gilette, N. M. , Langat, P. , Said, J. T. et al., SnapShot: COVID‐19. Cell. 2020. 181: 954–954.e1 32413300 10.1016/j.cell.2020.04.013 PMC7190493

[5] Riva, G. , Nasillo, V. , Tagliafico, E. , Trenti, T. , Comoli, P. and Luppi, M. , COVID‐19: more than a cytokine storm. Critical Care. 2020. 24: 549 32887652 10.1186/s13054-020-03267-w PMC7472946

[6] Fajgenbaum, D. C. , and June, C. H. Cytokine storm. New England Journal of Medicine. 2020. 383: 2255–2273. 33264547 10.1056/NEJMra2026131 PMC7727315

[7] Ellinghaus, D. , Degenhardt, F. , Bujanda, L. , Buti, M. , Albillos, A. , Invernizzi, P. , Fernández, J. et al., Genomewide association study of severe COVID‐19 with respiratory failure. New England Journal of Medicine. 2020. 383: 1522–1534 32558485 10.1056/NEJMoa2020283 PMC7315890

[8] Zhang, Q. , Liu, Z. , Moncada‐Velez, M. , Chen, J. , Ogishi, M. , Bigio, B. , Yang, R. et al., Inborn errors of type I IFN immunity in patients with life‐threatening COVID‐19. Science. 2020. 370. 10.1126/science.abd4570 PMC7857407 32972995

[9] Zhang, X. , Tan, Y. , Ling, Y. , Lu, G. , Liu, F. , Yi, Z. , Jia, X. et al., Viral and host factors related to the clinical outcome of COVID‐19. Nature. 2020. 583: 437–440 32434211 10.1038/s41586-020-2355-0

[10] Van Der Made, C. I. , Simons, A. , Schuurs‐Hoeijmakers, J. , Van Den Heuvel, G. , Mantere, T. , Kersten, S. , Van Deuren, R. C. et al., Presence of genetic variants among young men with severe COVID‐19. JAMA–Journal of the American Medical Association. 2020. 324: 663–673 10.1001/jama.2020.13719 PMC7382021 32706371

[11] Schulte‐Schrepping, J. , Reusch, N. , Paclik, D. , Baßler, K. , Schlickeiser, S. , Zhang, B. , Krämer, B. et al., Severe COVID‐19 is marked by a dysregulated myeloid cell compartment. Cell. 2020. 182: 1419–1440.e23 32810438 10.1016/j.cell.2020.08.001 PMC7405822

[12] Bellmann‐Weiler, R. , Lanser, L. , Burkert, F. , Seiwald, S. , Fritsche, G. , Wildner, S. , Schroll, A. et al., Neopterin predicts disease severity in hospitalized patients with COVID‐19. Open Forum Infectious Diseases. 2021. 8: ofaa521 33442554 10.1093/ofid/ofaa521 PMC7665702

[13] Merad, M. and Martin, J. C. , Pathological inflammation in patients with COVID‐19: a key role for monocytes and macrophages. Nature Reviews Immunology. 2020. 20: 355–362 10.1038/s41577-020-0331-4 PMC7201395 32376901

[14] Laing, A. G. , Lorenc, A. , del Molino del Barrio, I. , Das, A. , Fish, M. , Monin, L. , Muñoz‐Ruiz, M. et al., A dynamic COVID‐19 immune signature includes associations with poor prognosis. Nature Medicine. 2020. 26: 1623–1635 10.1038/s41591-020-1038-6 32807934

[15] Lucas, C. , Wong, P. , Klein, J. , Castro, T. B. R. , Silva, J. , Sundaram, M. , Ellingson, M. K. et al., Longitudinal analyses reveal immunological misfiring in severe COVID‐19. Nature. 2020. 584: 463–469 32717743 10.1038/s41586-020-2588-y PMC7477538

[16] Christensen, E. E. , Jørgensen, M. J. , Nore, K. G. , Dahl, T. B. , Yang, K. , Ranheim, T. , Huse, C. et al., Critical COVID‐19 is associated with distinct leukocyte phenotypes and transcriptome patterns. Journal of Internal Medicine. 2021. 290:677–692 34080738 10.1111/joim.13310 PMC8242786

[17] Silvin, A. , Chapuis, N. , Dunsmore, G. , Goubet, A. G. , Dubuisson, A. , Derosa, L. , Almire, C. et al., Elevated calprotectin and abnormal myeloid cell subsets discriminate severe from mild COVID‐19. Cell. 2020. 182: 1401–1418.e18 32810439 10.1016/j.cell.2020.08.002 PMC7405878

[18] Penttilä, P. A. , Van Gassen, S. , Panovska, D. , Vanderbeke, L. , Van Herck, Y. , Quintelier, K. , Emmaneel, A. et al., High dimensional profiling identifies specific immune types along the recovery trajectories of critically ill COVID19 patients. Cellular and Molecular Life Sciences. 2021. 78: 3987–4002 33715015 10.1007/s00018-021-03808-8 PMC7955698

[19] Vitte, J. , Diallo, A. B. , Boumaza, A. , Lopez, A. , Michel, M. , Allardet‐Servent, J. , Mezouar, S. et al., A granulocytic signature identifies COVID‐19 and its severity. Journal of Infectious Diseases. 2020. 222: 1985–1996 32941618 10.1093/infdis/jiaa591 PMC7543529

[20] Bellmann‐Weiler, R. , Lanser, L. , Barket, R. , Rangger, L. , Schapfl, A. , Schaber, M. , Fritsche, G. et al., Prevalence and predictive value of anemia and dysregulated iron homeostasis in patients with COVID‐19 infection. Journal of Clinical Medicine. 2020. 9: 2429 10.3390/jcm9082429 PMC7464087 32751400

[21] Mueller, K. A. L. , Langnau, C. , Günter, M. , Pöschel, S. , Gekeler, S. , Petersen‐Uribe, Á. , Kreisselmeier, K. ‐. P. et al., Numbers and phenotype of non‐classical CD14dimCD16+ monocytes are predictors of adverse clinical outcome in patients with coronary artery disease and severe SARS‐CoV‐2 infection. Cardiovascular Research. 2021. 117: 224–239 33188677 10.1093/cvr/cvaa328 PMC7665325

[22] Trombetta, A. C. , Farias, G. B. , Gomes, A. M. C. , Godinho‐Santos, A. , Rosmaninho, P. , Conceição, C. M. , Laia, J. et al., Severe COVID‐19 recovery is associated with timely acquisition of a myeloid cell immune‐regulatory phenotype. Frontiers in Immunology. 2021. 12: 2346 10.3389/fimmu.2021.691725 PMC8265310 34248984

[23] Vasse, M. , Zuber, B. , Goubeau, L. , Ballester, M. C. , Roumier, M. , Delcominette, F. , Habarou, F. et al., A low level of CD16pos monocytes in SARS‐CoV‐2 infected patients is a marker of severity. Clinical Chemistry and Laboratory Medicine. 2021. 59: 1315–1322 33606928 10.1515/cclm-2020-1801

[24] Bost, P. , De Sanctis, F. , Canè, S. , Ugel, S. , Donadello, K. , Castellucci, M. , Eyal, D. et al., Deciphering the state of immune silence in fatal COVID‐19 patients. Nature Communications. 2021. 12. 10.1038/s41467-021-21702-6 PMC7935849 33674591

[25] Haschka, D. , Hoffmann, A. and Weiss, G . Iron in immune cell function and host defense. 2021. 115: 27–36 10.1016/j.semcdb.2020.12.005 33386235

[26] Recalcati, S. , Locati, M. and Cairo, G. , Systemic and cellular consequences of macrophage control of iron metabolism. Seminars in Immunology. 2012. 24: 393–398 23375134 10.1016/j.smim.2013.01.001

[27] Lanser, L. , Fuchs, D. , Kurz, K. and Weiss, G. , Physiology and inflammation driven pathophysiology of iron homeostasis—mechanistic insights into anemia of inflammation and its treatment. Nutrients. 2021. 13: 3732 34835988 10.3390/nu13113732 PMC8619077

[28] Wong, K. L. , Yeap, W. H. , Tai, J. J. Y. , Ong, S. M. , Dang, T. M. and Wong, S. C. , The three human monocyte subsets: implications for health and disease. Immunologic Research. 2012. 53: 41–57 22430559 10.1007/s12026-012-8297-3

[29] Haschka, D. , Tymoszuk, P. , Bsteh, G. , Petzer, V. , Berek, K. , Theurl, I. , Berger, T. et al., Expansion of neutrophils and classical and nonclassical monocytes as a hallmark in relapsing‐remitting multiple sclerosis. Frontiers in Immunology. 2020. 11: 594 32411125 10.3389/fimmu.2020.00594 PMC7202453

[30] Patel, A. A. , Zhang, Y. , Fullerton, J. N. , Boelen, L. , Rongvaux, A. , Maini, A. A. , Bigley, V. et al., The fate and lifespan of human monocyte subsets in steady state and systemic inflammation. Journal of Experimental Medicine. 2017. 214: 1913–1923 28606987 10.1084/jem.20170355 PMC5502436

[31] Gatti, A. , Radrizzani, D. , Viganò, P. , Mazzone, A. and Brando, B. , Decrease of non‐classical and intermediate monocyte subsets in severe acute SARS‐CoV‐2 infection. Cytometry Part A. 2020. 97: 887–890 10.1002/cyto.a.24188 PMC7404377 32654350

[32] Geanon, D. , Lee, B. , Gonzalez‐Kozlova, E. , Kelly, G. , Handler, D. , Upadhyaya, B. , Leech, J. et al., A streamlined whole blood CyTOF workflow defines a circulating immune cell signature of COVID‐19. Cytometry Part A. 2021. 99: 446–461 10.1002/cyto.a.24317 PMC8013522 33496367

[33] Zingaropoli, M. A. , Nijhawan, P. , Carraro, A. , Pasculli, P. , Zuccalà, P. , Perri, V. , Marocco, R. et al., Increased sCD163 and sCD14 plasmatic levels and depletion of peripheral blood pro‐inflammatory monocytes, myeloid and plasmacytoid dendritic cells in patients with severe COVID‐19 pneumonia. Frontiers in Immunology. 2021. 12: 147 10.3389/fimmu.2021.627548 PMC7993197 33777012

[34] Kohonen, T . Self‐organizing maps. Berlin, Heidelberg: Springer, 1995

[35] Vesanto, J. and Alhoniemi, E. , Clustering of the self‐organizing map. IEEE Transactions on Neural Networks. 2000. 11: 586–600 18249787 10.1109/72.846731

[36] Weiss, G. and Carver, P. L . Role of divalent metals in infectious disease susceptibility and outcome. 2018. 24: 16–23 10.1016/j.cmi.2017.01.018 28143784

[37] Yağcı, S. , Serin, E. , Acicbe, Ö. , Zeren, M. İ. and Odabaşı, M. S. , The relationship between serum erythropoietin, hepcidin, and haptoglobin levels with disease severity and other biochemical values in patients with COVID‐19. International Journal of Laboratory Hematology. 2021. 00: 1–10 10.1111/ijlh.13479 PMC8014125 33554466

[38] Brandtner, A. , Tymoszuk, P. , Nairz, M. , Lehner, G. F. , Fritsche, G. , Vales, A. , Falkner, A. et al., Linkage of alterations in systemic iron homeostasis to patients’ outcome in sepsis: a prospective study. Journal of Intensive Care. 2020. 8: 76 33014378 10.1186/s40560-020-00495-8 PMC7528491

[39] Zhou, C. , Chen, Y. , Ji, Y. , He, X. and Xue, D. , Increased serum levels of hepcidin and ferritin are associated with severity of COVID‐19. Medical Science Monitor. 2020. 26: e926178–e926181 32978363 10.12659/MSM.926178 PMC7526336

[40] Nairz, M. , Theurl, I. , Swirski, F. K. and Weiss, G . ‘Pumping iron’—how macrophages handle iron at the systemic, microenvironmental, and cellular levels. 2017. 469: 397–418 10.1007/s00424-017-1944-8 PMC5362662 28251312

[41] McInnes, L. , Healy, J. and Melville, J. U . Uniform manifold approximation and projection for dimension reduction. 2018. 10.48550/arXiv.1802.03426

[42] Haschka, D. , Petzer, V. , Kocher, F. , Tschurtschenthaler, C. , Schaefer, B. , Seifert, M. , Sopper, S. et al., Classical and intermediate monocytes scavenge non‐transferrin‐bound iron and damaged erythrocytes. JCI Insight. 2019. 4: e98867 10.1172/jci.insight.98867 PMC6538345 30996139

[43] Nemeth, E. , Tuttle, M. S. , Powelson, J. , Vaughn, M. B. , Donovan, A. , Ward, D. M. , Ganz, T. et al., Hepcidin regulates cellular iron efflux by binding to ferroportin and inducing its internalization. Science (New York, N.Y.). 2004. 306: 2090–2093 10.1126/science.1104742 15514116

[44] Drakesmith, H. and Prentice, A. , Viral infection and iron metabolism. Nature Reviews. Microbiology. 2008. 6: 541–552 18552864 10.1038/nrmicro1930

[45] Bourgoin, P. , Soliveres, T. , Barbaresi, A. , Loundou, A. , Belkacem, I. A. , Arnoux, I. , Bernot, D. et al., CD169 and CD64 could help differentiate bacterial from CoVID‐19 or other viral infections in the Emergency Department. Cytometry Part A. 2021. 99: 435–445 10.1002/cyto.a.24314 PMC8014466 33491921

[46] Meghraoui‐Kheddar, A. , Chousterman, B. G. , Guillou, N. , Barone, S. M. , Granjeaud, S. , Vallet, H. , Corneau, A. et al., Two new immature and dysfunctional neutrophil cell subsets define a predictive signature of sepsis useable in clinical practice. bioRxiv. 2020:2020.05.29.123992

[47] Sack, U. , CD64 expression by neutrophil granulocytes. Cytometry Part B: Clinical Cytometry. 2017. 92: 189–191 25522066 10.1002/cyto.b.21216

[48] Luo, Q. , Xiao, P. , Li, X. , Deng, Z. , Qing, C. , Su, R. , Xu, J. et al., Overexpression of CD64 on CD14++CD16‐ and CD14++CD16+ monocytes of rheumatoid arthritis patients correlates with disease activity. Experimental and Therapeutic Medicine. 2018. 16: 2703–2711 30210612 10.3892/etm.2018.6452 PMC6122586

[49] Yang, J. , Fang, P. , Yu, D. , Zhang, L. , Zhang, D. , Jiang, X. , Yang, W. Y. et al., Chronic kidney disease induces inflammatory CD40+ monocyte differentiation via homocysteine elevation and DNA hypomethylation. Circulation Research. 2016. 119: 1226–1241 27992360 10.1161/CIRCRESAHA.116.308750 PMC5176108

[50] Hamers, A. A. J. , Dinh, H. Q. , Thomas, G. D. , Marcovecchio, P. , Blatchley, A. , Nakao, C. S. , Kim, C. et al., Human monocyte heterogeneity as revealed by high‐dimensional mass cytometry. Arteriosclerosis, Thrombosis, and Vascular Biology. 2019. 39: 25–36 30580568 10.1161/ATVBAHA.118.311022 PMC6697379

[51] Ross, S. L. , Biswas, K. , Rottman, J. , Allen, J. R. , Long, J. , Miranda, L. P. , Winters, A. et al., Identification of antibody and small molecule antagonists of ferroportin‐hepcidin interaction. Frontiers in Pharmacology. 2017. 8: 838 29209212 10.3389/fphar.2017.00838 PMC5702341

[52] Cossarizza, A. , Chang, H. D. , Radbruch, A. , Abrignani, S. , Addo, R. , Akdis, M. , Andrä, I. ** *et al* **. Guidelines for the use of flow cytometry and cell sorting in immunological studies (third edition). European Journal of Immunology. 2021. 51: 2708–3145 34910301 10.1002/eji.202170126 PMC11115438

[53] Wickham, H. , Averick, M. , Bryan, J. , Chang, W. , McGowan, L. , François, R. , Grolemund, G. et al., Welcome to the tidyverse. Journal of Open Source Software. 2019. 4: 1686

[54] Wilke, C. O . Fundamentals of data visualization: a primer on making informative and compelling figures, 1st ed.. Sebastopol: O'Reilly Media, 2019. p. 389.

[55] Benjamini, Y. and Hochberg, Y. , Controlling the false discovery rate: a practical and powerful approach to multiple testing. Journal of the Royal Statistical Society: Series B (Methodological). 1995. 57: 289–300

