# Supplementary material for: Alterations of blood monocyte subset distribution and surface phenotype are linked to infection severity in COVID‐19 inpatients
Source: Eur J Immunol. 2022 May 6;52(8):1285–96. doi: 10.1002/eji.202149680 (PMC9348104; doi:10.1002/eji.202149680)
Supplement: Supplementary file 1 — Supporting Information [file EJI-52-1285-s002.pdf]

Alterations of blood monocyte subset distribution and surface phenotype are linked to infection severity in COVID-19 inpatients

Supplementary Material

Supplementary Tables

Supplementary Table S1: Characteristic of the healthy and COVID-19 study participants.

Variable	Healthy	Moderate COVID-19	Severe COVID-19	Comparison: all groups	Comparison: COVID-19
N participants	7	16	32		
Age, years	Mean = 37 (SD: 8.4) Median = 36 [IQR: 32 - 42] Range: 27 - 49	Mean = 48 (SD: 18) Median = 46 [IQR: 38 - 60] Range: 18 - 78	Mean = 69 (SD: 13) Median = 71 [IQR: 64 - 79] Range: 34 - 90	$p < 0.001$[2]	$p < 0.001$[3]
Sex	female: 43% (n = 3) male: 57% (n = 4)	female: 44% (n = 7) male: 56% (n = 9)	female: 28% (n = 9) male: 72% (n = 23)	ns ($p = 0.5$)[4]	ns ($p = 0.45$)[4]
BMI, kg/m^2[1]	Mean = 24 (SD: 3.2) Median = 23 [IQR: 22 - 24] Range: 20 - 30	Mean = 26 (SD: 5.1) Median = 25 [IQR: 23 - 29] Range: 18 - 37	Mean = 28 (SD: 6.3) Median = 26 [IQR: 23 - 33] Range: 18 - 44	ns ($p = 0.16$)[2]	ns ($p = 0.35$)[3]
Length of hospital stay, days	Mean = 0 (SD: 0) Median = 0 [IQR: 0 - 0] Range: 0 - 0	Mean = 7.7 (SD: 5.5) Median = 6 [IQR: 3.8 - 11] Range: 1 - 19	Mean = 19 (SD: 26) Median = 12 [IQR: 9 - 16] Range: 4 - 140		$p = 0.011$[3]
Oxygen therapy	0% (n = 0)	0% (n = 0)	100% (n = 32)		
ICU stay	0% (n = 0)	0% (n = 0)	22% (n = 7)		
Mortality	0% (n = 0)	0% (n = 0)	6.2% (n = 2)		

[1]Body Mass Index

[2]Kruskal-Wallis test

[3]Mann-Whitney test

[4]χ^2 test

Supplementary Table S2: Antibodies used for flow cytometry staining.

Staining type	Antigen	Clone	Fluorophore
backbone	HLA-DR	G46-6	BV421
	CD14	MφP9	BB700
	CD16	3G8	BV605
	CD45	HI30	BV510
	CD3	UCHT1	PE-Cy5
	CD19	HIB19	PE-Cy5
	CD56	B159	PE-Cy5
	CD11b	M1/70	BB515
	CD15	HI98	Alexa 700
	CCR2	K036C2	PE-Cy7
	CD62L	DREG-56	APC-Fire750
test	Isotype Rat	RTK2758	Alexa647
	FPN1	38G6	Alexa647
	CD71	OKT9	Alexa647
	CD163	GHI/61	Alexa647
	CD40	5C3	Alexa647
	CD80	2D10	Alexa647
	CD86	IT2.2	Alexa647
	CD64	10.1	Alexa647
	Isotype Rat	X40	BV650
	Isotype Mouse	P3.6.2.8.1	PE-eFluor610
	CD274	MIH1	BV650
	CD279	J105	PE-eFluor610

Supplementary Table S3: Variables obtained from cytometry staining analyzed in the study.

**Variable**	**Unit[1]**
Neutrophil percent	% of CD45+
Monocyte percent	% of CD45+
Classical monocyte percent	% of CD45+
Classical monocyte percent	% of panMono
Intermediate monocyte percent	% of CD45+
Intermediate monocyte percent	% of panMono
Non-classical monocyte percent	% of CD45+
Non-classical monocyte percent	% of panMono
Neutrophil CD274	ΔMFI
Neutrophil CD279	ΔMFI
Class. monocyte CD274	ΔMFI
Class. monocyte CD279	ΔMFI
Int. monocyte CD274	ΔMFI
Int. monocyte CD279	ΔMFI
Non-class. monocyte CD274	ΔMFI
Class. monocyte CD163	ΔMFI
Int. monocyte CD163	ΔMFI
Non-class. monocyte CD163	ΔMFI
Neutrophil CD40	ΔMFI
Class. monocyte CD40	ΔMFI
Int. monocyte CD40	ΔMFI
Non-class. monocyte CD40	ΔMFI
Neutrophil CD64	ΔMFI
Class. monocyte CD64	ΔMFI

Variable	Unit[1]
Int. monocyte CD64	ΔMFI
Non-class. monocyte CD64	ΔMFI
Class. monocyte CD71	ΔMFI
Int. monocyte CD71	ΔMFI
Non-class. monocyte CD71	ΔMFI
Neutrophil CD86	ΔMFI
Class. monocyte CD86	ΔMFI
Int. monocyte CD86	ΔMFI
Non-class. monocyte CD86	ΔMFI
Neutrophil FPN1	ΔMFI
Class. monocyte FPN1	ΔMFI
Int. monocyte FPN1	ΔMFI
Non-class. monocyte FPN1	ΔMFI
Monocyte: Lymphocyte Ratio	
Neutrophil: Lymphocyte Ratio	
Lin-	% of CD45+

[1]panMono: monocyte cluster cells defined by UMAP, ΔMFI: difference in median fluorescence intensity between the test antibody and isotype-stained sample

Supplementary Table S4: Characteristic of COVID-19 patients assigned to the participant clusters defined by flow cytometry features.

Variable	Cluster #1	Cluster #2	Cluster #3	Cluster #4	Comparison: all groups	Comaprison: Cluster #1
N COVID-19 patients	9	12	11	9		
Age, years	Mean = 61 (SD: 17)<br>Median = 68 [IQR: 46 - 75]<br>Range: 35 - 79<br>Complete: n = 9	Mean = 58 (SD: 18)<br>Median = 64 [IQR: 44 - 72]<br>Range: 18 - 79<br>Complete: n = 12	Mean = 61 (SD: 20)<br>Median = 69 [IQR: 58 - 73]<br>Range: 24 - 79<br>Complete: n = 11	Mean = 61 (SD: 18)<br>Median = 58 [IQR: 46 - 79]<br>Range: 34 - 85<br>Complete: n = 9	ns (p = 0.99)[2]	#2: ns (p = 0.93)<br>#3: ns (p = 0.97)<br>#4: ns (p = 1)[3]
Sex	female: 22% (n = 2)<br>male: 78% (n = 7)<br>Complete: n = 9	female: 50% (n = 6)<br>male: 50% (n = 6)<br>Complete: n = 12	female: 45% (n = 5)<br>male: 55% (n = 6)<br>Complete: n = 11	female: 22% (n = 2)<br>male: 78% (n = 7)<br>Complete: n = 9	ns (p = 0.57)[4]	#2: ns (p = 0.8)<br>#3: ns (p = 0.73)<br>#4: ns (p = 1)[4]
BMI, kg/m^2[1]	Mean = 26 (SD: 3.5)<br>Median = 25 [IQR: 23 - 30]<br>Range: 22 - 30<br>Complete: n = 9	Mean = 28 (SD: 8.8)<br>Median = 24 [IQR: 22 - 36]<br>Range: 18 - 44<br>Complete: n = 12	Mean = 27 (SD: 4.7)<br>Median = 26 [IQR: 24 - 28]<br>Range: 19 - 35<br>Complete: n = 11	Mean = 26 (SD: 4.9)<br>Median = 26 [IQR: 23 - 28]<br>Range: 20 - 35<br>Complete: n = 8	ns (p = 0.99)[2]	#2: ns (p = 1)<br>#3: ns (p = 0.88)<br>#4: ns (p = 1)[3]
Length of hospital stay, days	Mean = 11 (SD: 5.7)<br>Median = 12 [IQR: 8 - 14]<br>Range: 2 - 19<br>Complete: n = 9	Mean = 24 (SD: 40)<br>Median = 11 [IQR: 6 - 20]<br>Range: 4 - 140<br>Complete: n = 11	Mean = 12 (SD: 7)<br>Median = 11 [IQR: 7.8 - 13]<br>Range: 4 - 29<br>Complete: n = 10	Mean = 17 (SD: 20)<br>Median = 9 [IQR: 9 - 11]<br>Range: 4 - 67<br>Complete: n = 9	ns (p = 0.99)[2]	#2: ns (p = 0.93)<br>#3: ns (p = 0.97)<br>#4: ns (p = 1)[3]

Variable	Cluster #1	Cluster #2	Cluster #3	Cluster #4	Comparison: all groups	Comaprison: Cluster #1
Oxygen therapy	44% (n = 4) Complete: n = 9	58% (n = 7) Complete: n = 12	73% (n = 8) Complete: n = 11	89% (n = 8) Complete: n = 9	ns (p = 0.37)[4]	#2: ns (p = 0.93) #3: ns (p = 0.7) #4: ns (p = 0.39)[4]
ICU stay	0% (n = 0) Complete: n = 9	25% (n = 3) Complete: n = 12	18% (n = 2) Complete: n = 11	11% (n = 1) Complete: n = 9	ns (p = 0.57)[4]	#2: ns (p = 0.77) #3: ns (p = 0.73) #4: ns (p = 1)[4]
IL6, pg/mL	Mean = 27 (SD: 41) Median = 8.3 [IQR: 2.7 - 25] Range: 1.5 - 120 Complete: n = 9	Mean = 24 (SD: 29) Median = 8.4 [IQR: 3.2 - 36] Range: 1.5 - 76 Complete: n = 12	Mean = 41 (SD: 25) Median = 28 [IQR: 23 - 45] Range: 21 - 89 Complete: n = 11	Mean = 47 (SD: 55) Median = 16 [IQR: 5.2 - 77] Range: 2.4 - 160 Complete: n = 9	ns (p = 0.27)[2]	#2: ns (p = 0.93) #3: ns (p = 0.14) #4: ns (p = 0.57)[3]
CRP, mg/L	Mean = 1.9 (SD: 2.6) Median = 0.33 [IQR: 0.21 - 2.8] Range: 0.06 - 8.1 Complete: n = 9	Mean = 3.2 (SD: 3.6) Median = 1.1 [IQR: 0.69 - 5.4] Range: 0.06 - 11 Complete: n = 12	Mean = 5.3 (SD: 3.2) Median = 3.2 [IQR: 2.7 - 7.5] Range: 2.4 - 11 Complete: n = 11	Mean = 6 (SD: 4.4) Median = 7.5 [IQR: 1.5 - 9.4] Range: 0.77 - 12 Complete: n = 9	ns (p = 0.14)[2]	#2: ns (p = 0.77) #3: p = 0.05 #4: ns (p = 0.13)[3]
Neopterin, nmol/L	Mean = 21 (SD: 23) Median = 13 [IQR: 6.3 - 29] Range: 4.1 - 74 Complete: n = 9	Mean = 75 (SD: 110) Median = 39 [IQR: 28 - 51] Range: 16 - 410 Complete: n = 11	Mean = 44 (SD: 11) Median = 40 [IQR: 39 - 43] Range: 35 - 72 Complete: n = 9	Mean = 52 (SD: 28) Median = 53 [IQR: 33 - 61] Range: 18 - 100 Complete: n = 9	ns (p = 0.14)[2]	#2: ns (p = 0.15) #3: p = 0.05 #4: ns (p = 0.13)[3]

Variable	Cluster #1	Cluster #2	Cluster #3	Cluster #4	Comparison: all groups	Comaprison: Cluster #1
Ferritin, ng/mL	Mean = 410 (SD: 330) Median = 240 [IQR: 180 - 760] Range: 88 - 950 Complete: n = 9	Mean = 320 (SD: 340) Median = 200 [IQR: 140 - 330] Range: 29 - 1200 Complete: n = 12	Mean = 640 (SD: 380) Median = 530 [IQR: 350 - 880] Range: 230 - 1200 Complete: n = 11	Mean = 690 (SD: 560) Median = 500 [IQR: 400 - 900] Range: 91 - 2000 Complete: n = 9	ns (p = 0.22)[2]	#2: ns (p = 0.8) #3: ns (p = 0.26) #4: ns (p = 0.52)[3]
Iron, μM	Mean = 12 (SD: 7.4) Median = 10 [IQR: 6.8 - 16] Range: 4 - 27 Complete: n = 9	Mean = 7.1 (SD: 5.6) Median = 4.6 [IQR: 3.6 - 7.9] Range: 2.3 - 20 Complete: n = 12	Mean = 5.2 (SD: 2.6) Median = 4.6 [IQR: 3.2 - 6.4] Range: 2.2 - 9.9 Complete: n = 11	Mean = 7.7 (SD: 7.3) Median = 4.5 [IQR: 3 - 9.4] Range: 1.3 - 21 Complete: n = 9	ns (p = 0.27)[2]	#2: ns (p = 0.38) #3: p = 0.05 #4: ns (p = 0.39)[3]
TF-Sat, %	Mean = 25 (SD: 14) Median = 21 [IQR: 17 - 31] Range: 8 - 52 Complete: n = 9	Mean = 14 (SD: 11) Median = 9.5 [IQR: 7 - 14] Range: 4 - 40 Complete: n = 12	Mean = 14 (SD: 8.8) Median = 9 [IQR: 7 - 18] Range: 5 - 30 Complete: n = 11	Mean = 16 (SD: 13) Median = 12 [IQR: 7 - 20] Range: 4 - 39 Complete: n = 9	ns (p = 0.27)[2]	#2: ns (p = 0.16) #3: ns (p = 0.1) #4: ns (p = 0.37)[3]

[1]Body Mass Index

[2]Kruskal-Wallis test

[3]Mann-Whitney test

[4]χ^2 test

Supplementary Table S5: Complete study dataset. The table is available as a supplementary Excel file.

Supplementary Figures

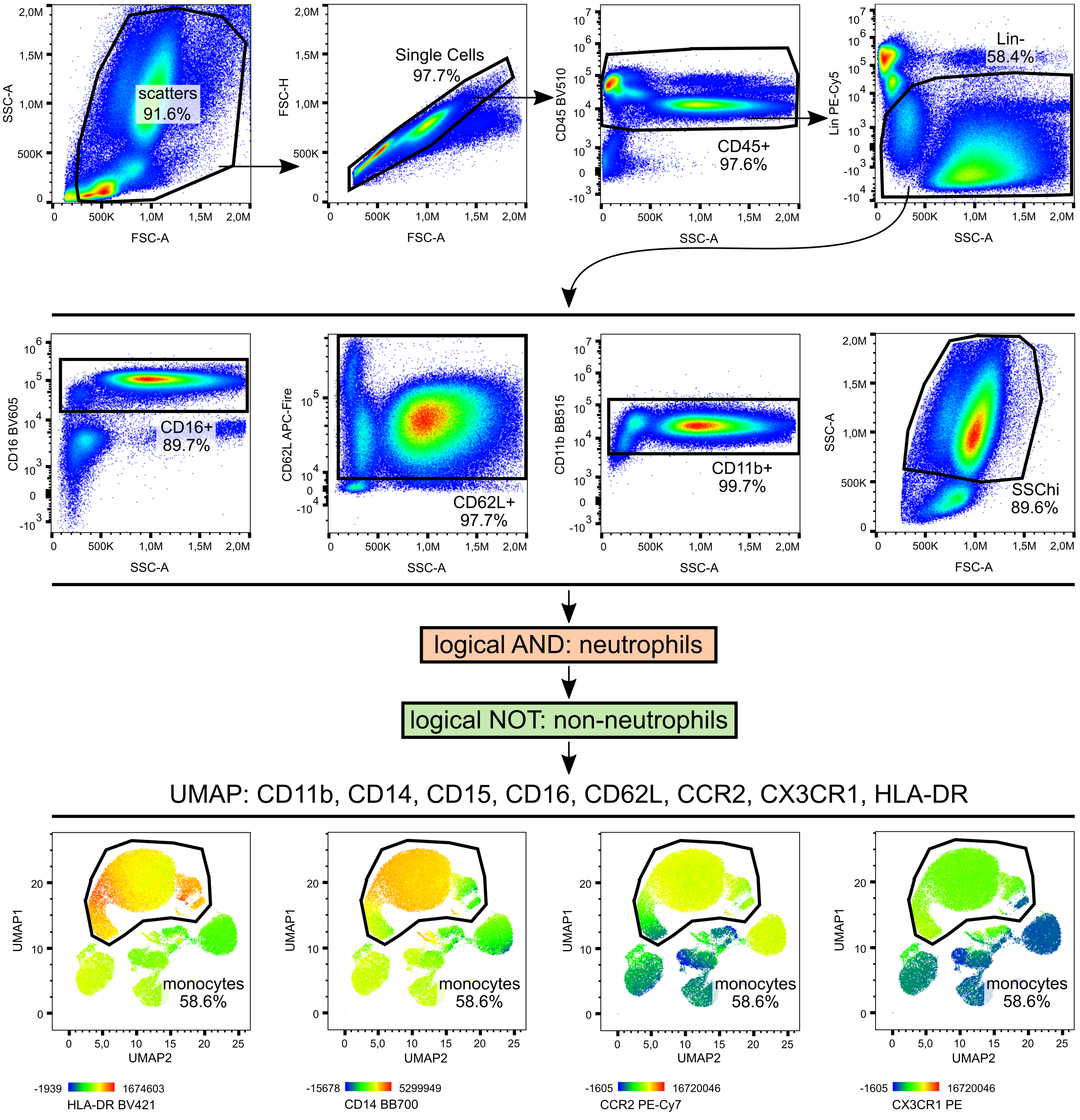

Supplementary Figure S1. Gating strategy and identification of blood neutrophils and monocytes.

Lin: lineage staining (CD3, CD19, CD56). Neutrophils were identified within the $CD45^+$ Lin^- blood leukocyte subset by logical gating (AND) of $CD16^+$, $CD62L^+$, $CD11b^+$ and SSC^{hi} events.

To identify monocytes, the non-neutrophil cells (NOT logical gate) were subjected to UMAP (uniform manifold approximation and projection, euclidean distance, k = 9 nearest neighbors, distance cutoff = 0.5) in respect to HLA-DR, CD11b, CD14, CD16, CCR2, CX3CR1, CD62L and CD15 signals. The monocyte cluster was distinguished by high expression of HLA-DR, CD14, CCR2 and CX3CR1. Representative flow cytometry analysis results for one healthy blood donor are presented.

Monocyte cluster:

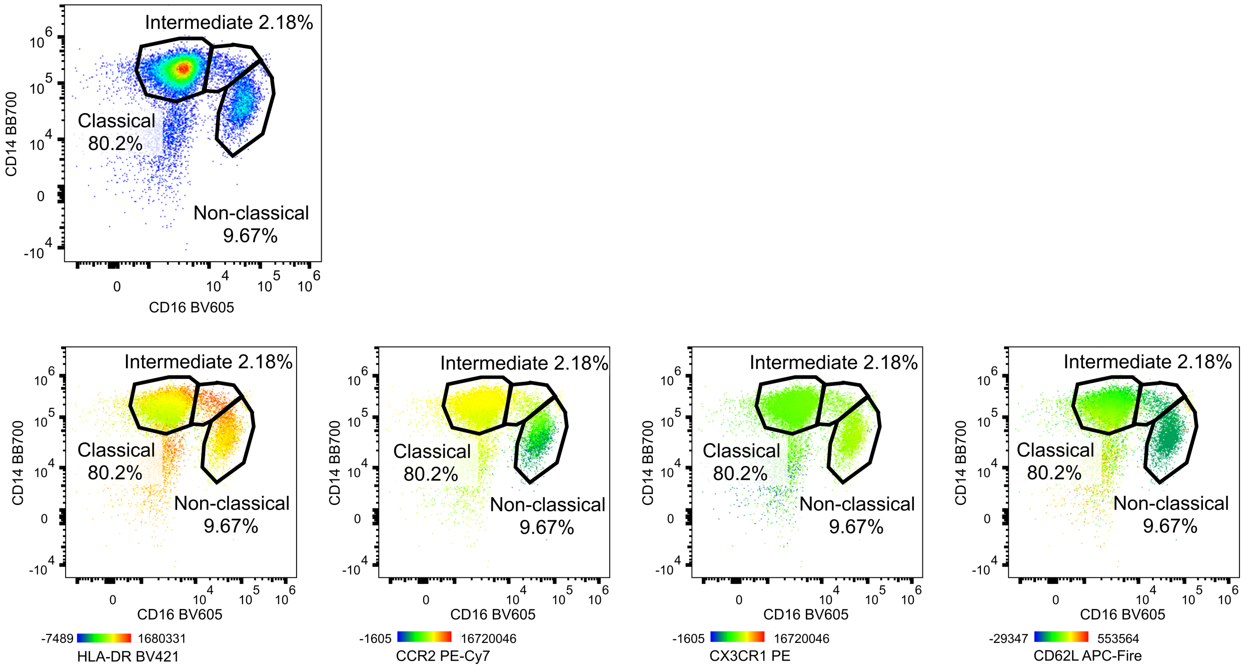

Supplementary Figure S2. Identification of blood monocyte subsets.

Monocyte cluster cells were identified as presented in **Supplementary Figure S1**. Classical monocytes were defined as CD14^{hi} CD16$^{-/lo}$ CCR2^{hi} HLA-DR^{+} CX3CR1^{lo} monocyte cluster cells. Intermediate monocytes were defined as CD14$^{int/hi}$ CD16^{+} CCR2^{int} HLA-DR^{bright} CX3CR1^{lo} monocyte cluster cells. Non-classical monocytes were defined as CD14^{lo} Cd16^{+} CCR2^{low} HLA-DR^{+} CX3CR1^{+} monocyte cluster cells. Representative flow cytometry analysis results for one healthy blood donor are presented.

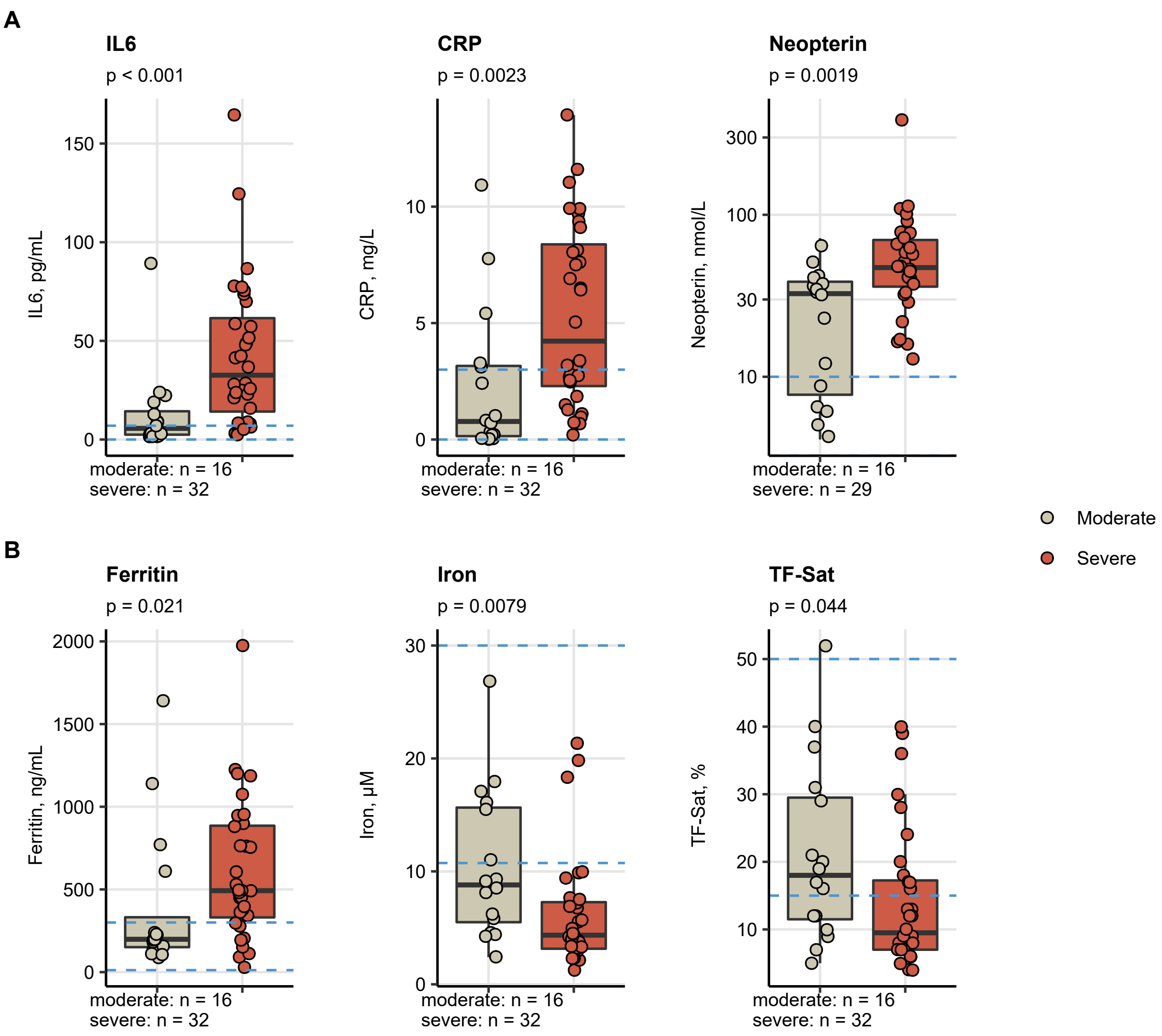

Supplementary Figure S3. Systemic inflammation and iron turnover markers in hospitalized COVID-19 subjects.

Markers of systemic inflammation (**A**): C-reactive protein (CRP), interleukin-6 (IL6), neopterin, and iron turnover (**B**): ferritin, iron and transferrin saturation (TF-Sat), were determined in plasma of moderate and severe COVID-19 study participants at hospital admission. Statistical significance was determined by Mann-Whitney U test with Benjamini-Hochberg adjustment for multiple testing. P values are indicated in the plot sub-heading. Each point represents a single observation, boxes represent medians with interquartile range (IQR), whiskers span over the 150% IQR range. Blue dashed lines represent the normal range of the parameter. N = 45 - 48 biological replicates (blood cell donors) per inflammatory parameter. Numbers of moderate and severe COVID-19 blood cell donors are indicated under each plot.

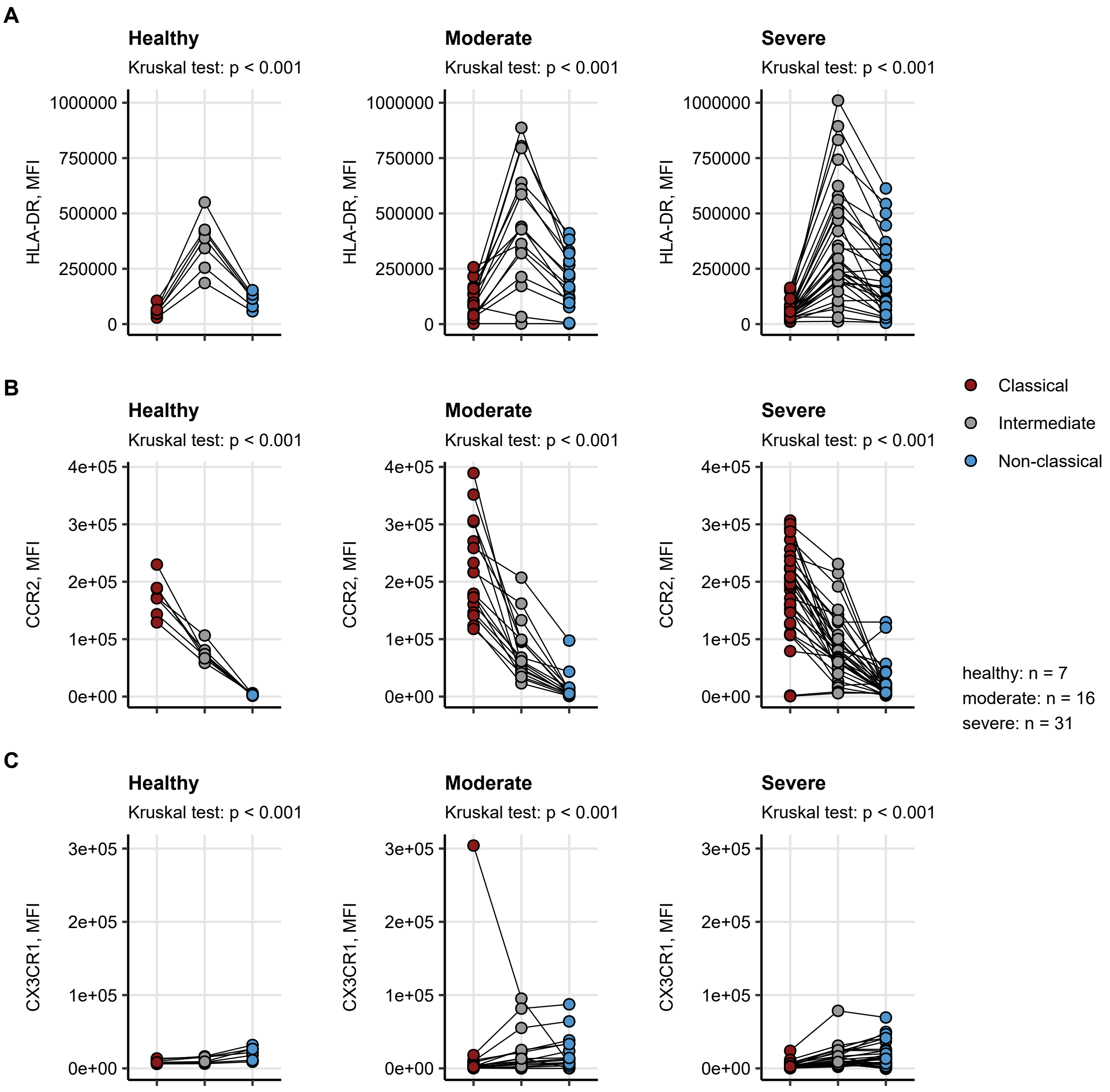

Supplementary Figure S4. Regulation of the monocyte subset markers HLA-DR, CCR2 and CX3CR1 in healthy controls, moderate and severe COVID-19.

Surface expression of HLA-DR (**A**), CCR2 (**B**) and CX3CR1 (**C**) in classical, intermediate and non-classical monocytes (**Supplementary Figure S2**) was measured as mean fluorescence intensity (MFI) in healthy controls, moderate and severe COVID-19 patients. Statistical significance of the expression differences between the monocyte subsets was determined with Friedman test (grouping factor: cell donor) with Benjamini-Hochberg adjustment for multiple testing. P values are indicated in the plot sub-heading. Each point represents a

single observation, gray lines connect values obtained from the same cell donor. N = 54 biological replicates (blood cell donors, healthy: n = 7, moderate COVID-19: n = 16, severe COVID-19: n = 31).

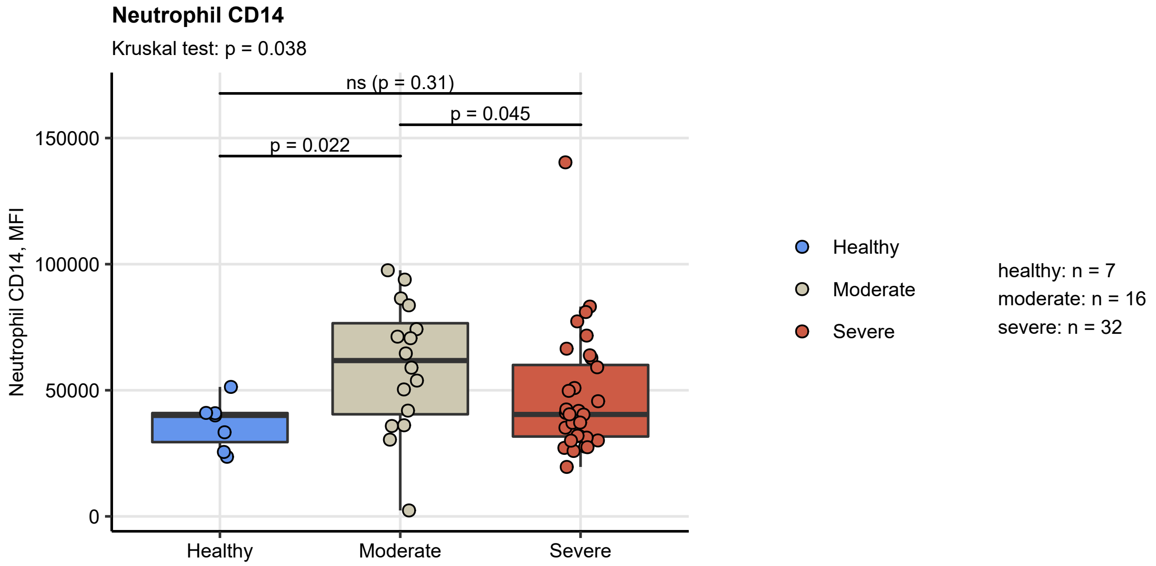

Supplementary Figure S5. Regulation of neutrophil CD14 in healthy controls, moderate and severe COVID-19.

Surface expression of neutrophil CD14 (**Supplementary Figure S1**) was measured as mean fluorescence intensity (MFI) in healthy controls, moderate and severe COVID-19 patients. Statistical significance was determined by Kruskal-Wallis test with Mann-Whitney post-hoc test. Testing results were adjusted for multiple comparisons with Benjamini-Hochberg method. Kruskal-Wallis p values are indicated in the plot sub-heading, post-hoc test results are shown in the plot. Each point represents a single observation, boxes represent medians with interquartile range (IQR), whiskers span over the 150% IQR range. N = 55 biological replicates (blood cell donors, healthy: n = 7, moderate COVID-19: n = 16, severe COVID-19: n = 32).

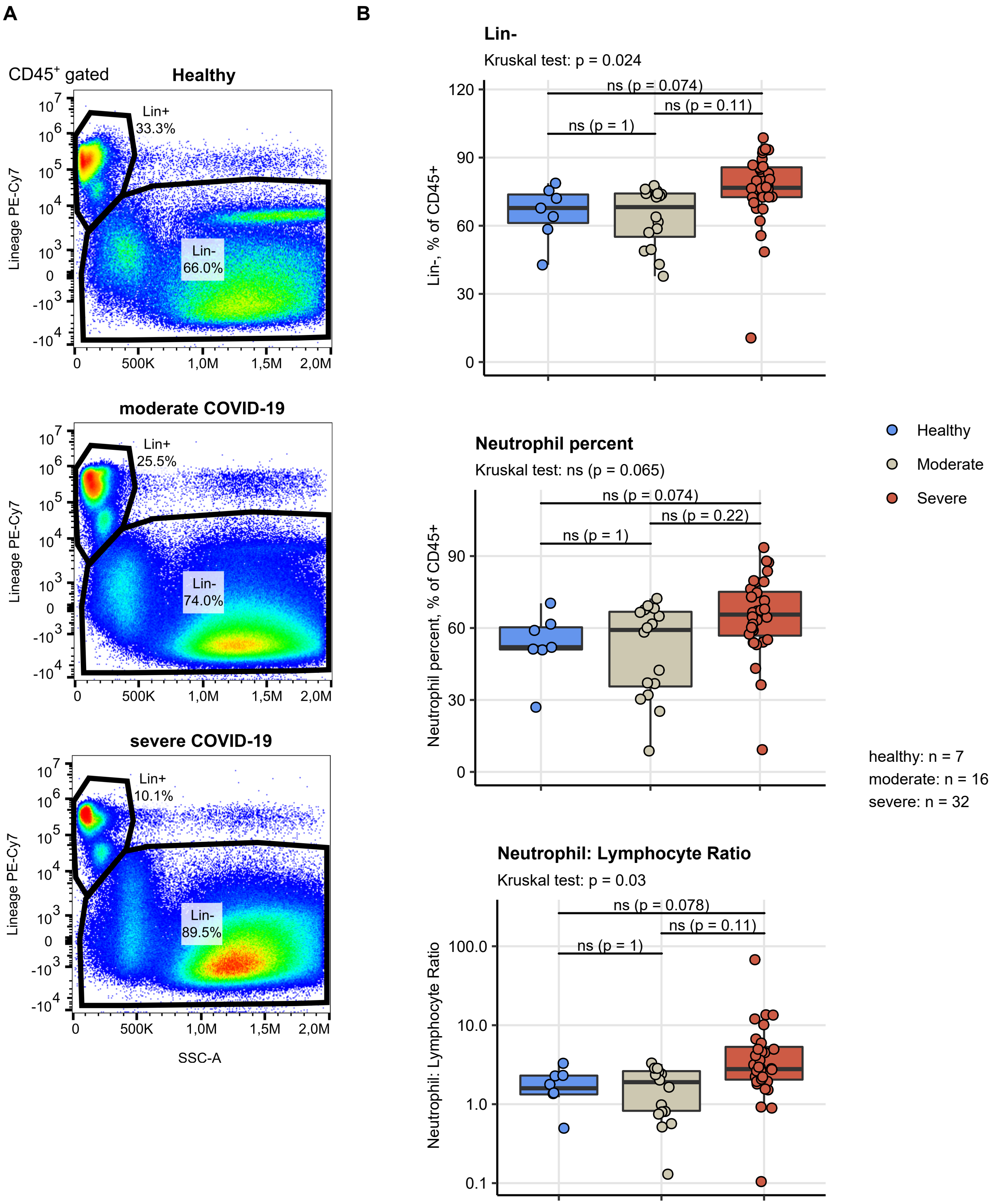

Supplementary Figure S6. Cytometry markers of myeloid leukocyte expansion in healthy controls, moderate and severe COVID-19.

Percentages of lineage-negative cells (Lin^-) and neutrophils within the $CD45^+$ leukocyte compartment and neutrophil:leukocyte ratio (**Supplementary Figure S1**) were measured in healthy controls, moderate and severe COVID-19 patients. Statistical significance was determined by Kruskal-Wallis test with Mann-Whitney post-hoc test. Testing results were adjusted for multiple comparisons with Benjamini-Hochberg method.

(A) Representative cytometry result of one healthy, one moderate COVID-19 and one severe COVID blood cell donor. $CD45^+$ cells are presented.

(B) Summary plots. Kruskal-Wallis p values are indicated in the plot sub-heading, post-hoc test results are shown in the plot, numbers of complete observations are presented next to the plot. Each point represents a single observation, boxes represent medians with interquartile range (IQR), whiskers span over the 150% IQR range. N = 55 biological replicates (blood cell donors, healthy: n = 7, moderate COVID-19: n = 16, severe COVID-19: n = 32).

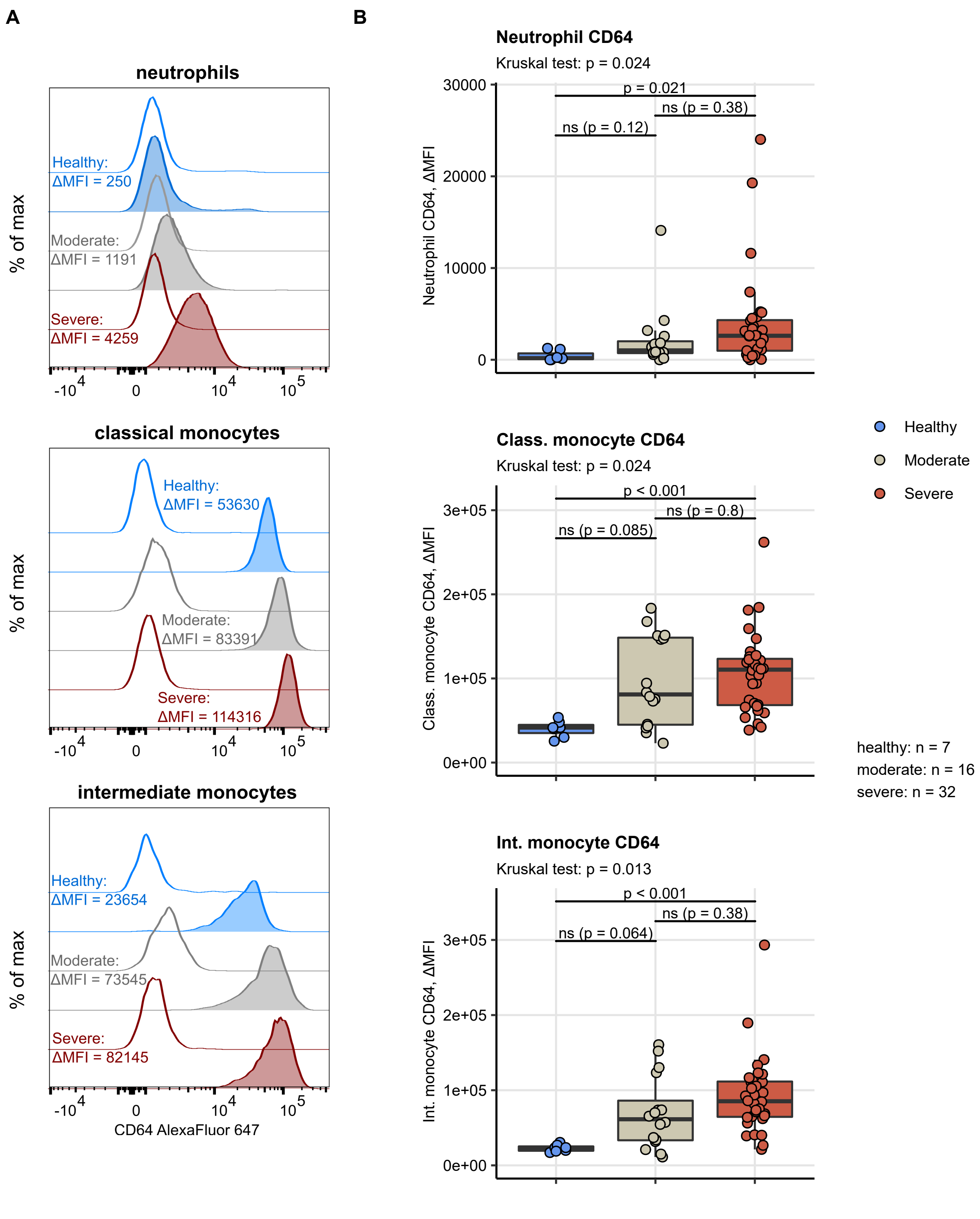

Supplementary Figure S7. Regulation of myeloid leukocyte CD64 in healthy controls, moderate and severe COVID-19.

Surface expression of CD64 was measured as delta median fluorescence intensity (ΔMFI) versus isotype staining in neutrophils, classical and intermediate monocytes (**Supplementary Figure S1** - **S2**) in healthy controls, moderate and severe COVID-19 patients. Statistical significance was determined by Kruskal-Wallis test with Mann-Whitney post-hoc test. Testing results were adjusted for multiple comparisons with Benjamini-Hochberg method.

(A) Representative cytometry result of one healthy, one moderate COVID-19 and one severe COVID blood cell donor. Open histograms: isotype, tinted histograms: specific staining.

(B) Summary plots. Kruskal-Wallis p values are indicated in the plot sub-heading, post-hoc test results are shown in the plot, numbers of complete observations are presented next to the plot. Each point represents a single observation, boxes represent medians with interquartile range (IQR), whiskers span over the 150% IQR range. N = 55 biological replicates (blood cell donors, healthy: n = 7, moderate COVID-19: n = 16, severe COVID-19: n = 32).

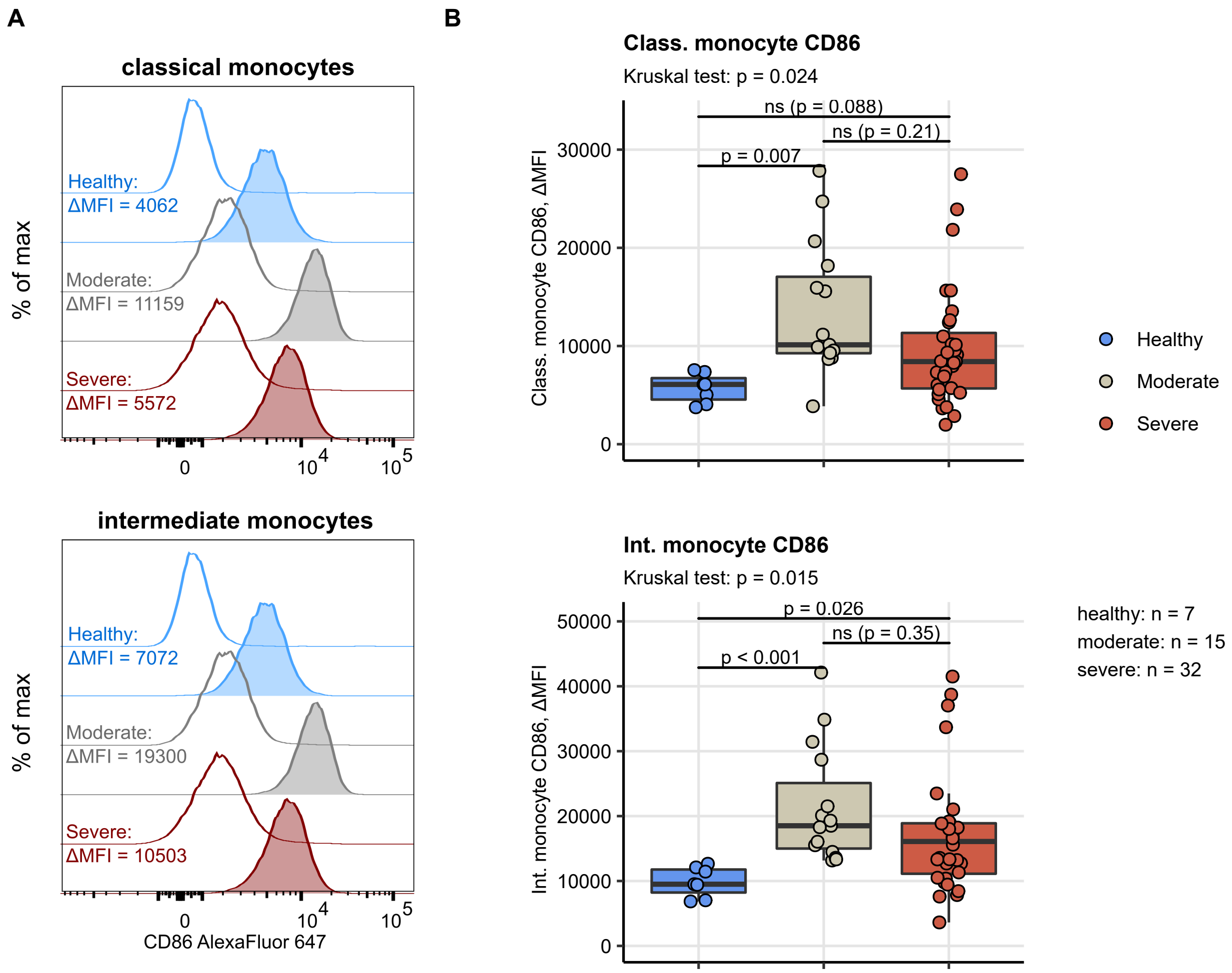

Supplementary Figure S8. Regulation of monocyte CD86 in healthy controls, moderate and severe COVID-19.

Surface expression of CD86 was measured as delta median fluorescence intensity (*Δ*MFI) versus isotype staining in classical and intermediate monocytes (**Supplementary Figure S2**) in healthy controls, moderate and severe COVID-19 patients. Statistical significance was determined by Kruskal-Wallis test with Mann-Whitney post-hoc test. Testing results were adjusted for multiple comparisons with Benjamini-Hochberg method.

(A) Representative cytometry result of one healthy, one moderate COVID-19 and one severe COVID blood cell donor. Open histograms: isotype, tinted histograms: specific staining.

(B) Summary plots. Kruskal-Wallis p values are indicated in the plot sub-heading, post-hoc test results are shown in the plot, numbers of complete observations are presented next to the plot. Each point represents a single observation, boxes represent medians with

interquartile range (IQR), whiskers span over the 150% IQR range. N = 54 biological replicates (blood cell donors, healthy: n = 7, moderate COVID-19: n = 15, severe COVID-19: n = 32).

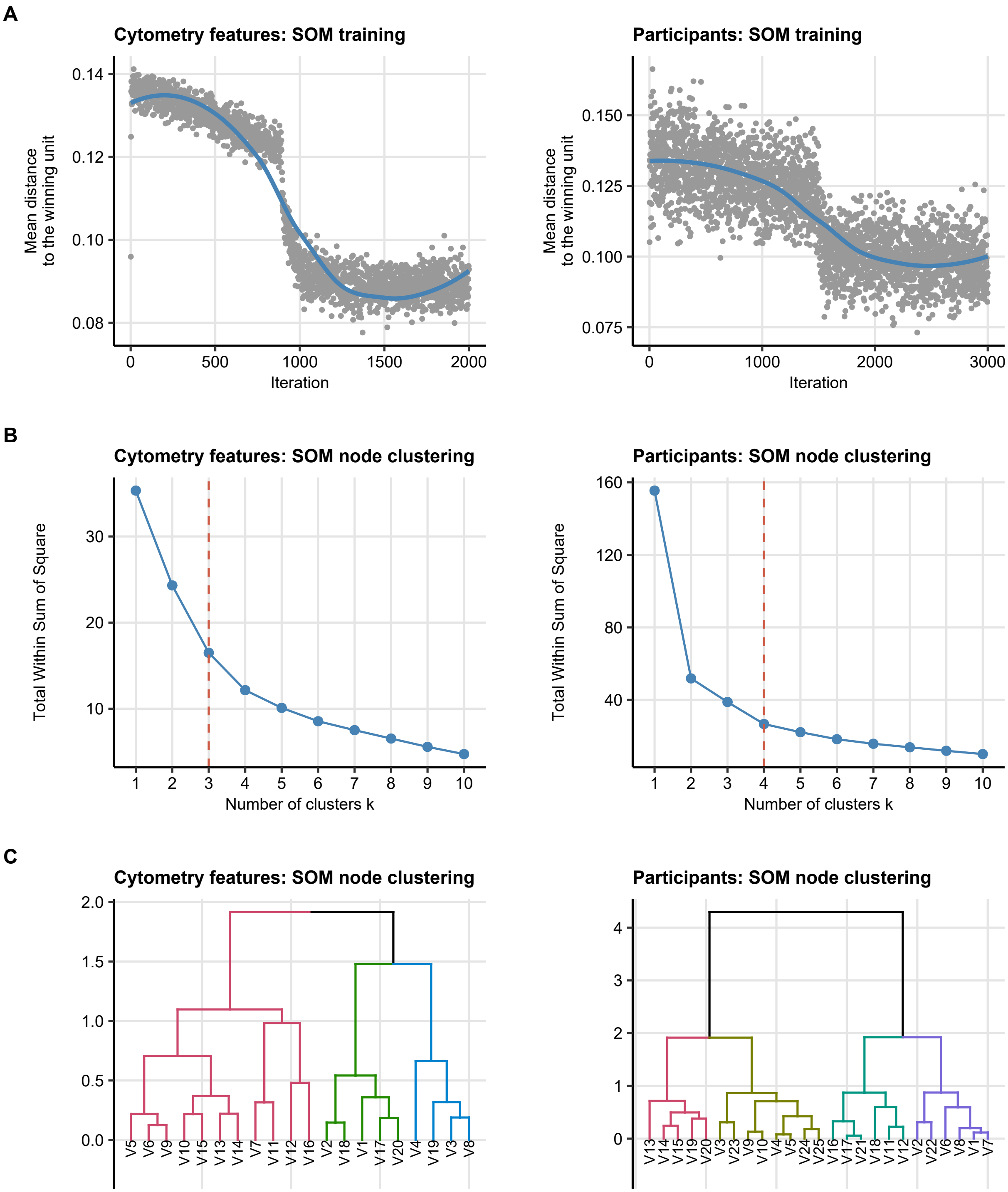

Supplementary Figure S9. Training and clustering of self-organizing maps.

Flow cytometry parameters (**Supplementary Table S3**) and study participants were subjected to self-organizing map (SOM) dimensionality reduction (5 × 5 hexagonal grid, cosine distance between the observations) followed by SOM node clustering with Ward D2 algorithm (cosine distance between the nodes). N = 48 biological replicates (blood cell donors).

(A) SOM training process for the cytometry parameters and study participants. Mean distance to the SOM winning unit as a function of algorithm iteration is presented. Each point represents a single iteration blue lines depict LOESS (locally weighted scatterplot smoothing) trends.

(B) Clustering of the SOM nodes for the cytometry parameters and study participants. Total within cluster sum-of-squares are shown as a function of cluster numbers. Red lines indicate the selected optimal number of clusters.

(C) Clustering of the SOM nodes for the cytometry parameters and study participants. Node clustering dendrograms are presented.
